# Psychological well-being during the COVID-19 pandemic: Combining a web survey with experience sampling methodology

**DOI:** 10.1371/journal.pone.0282649

**Published:** 2023-03-24

**Authors:** Yury Shevchenko, Noemi Huber, Ulf-Dietrich Reips

**Affiliations:** Research Methods, Assessment, and iScience, Department of Psychology, University of Konstanz, Konstanz, Germany; Xiamen University, CHINA

## Abstract

COVID-19-related regulations have impacted the economy and people’s well-being, highlighting the long-standing problem of inequality. This research explored how COVID-19-related restrictive policies, such as a lockdown or social distancing, affected people’s well-being. In Study 1, a cross-sectional online survey (*N* = 685), we examined the associations between socio-economic characteristics, the number of resources, their relative change, people’s stress levels, and their support of restrictive policies. We found that financial loss due to COVID-19, the number of children at home, and the intensity of restrictive measures were associated with higher stress by restrictive measures. The lower support for restrictive measures was observed among those who experienced financial loss due to COVID-19, had more children at home, less frequently accessed COVID-19-related information in the media, and did not perform self-isolation. Men were generally less supportive of restrictions than women, and the number of new COVID-19 cases was negatively related to the support. Lower stress and higher support for restrictive measures were positively associated with life satisfaction. In Study 2, an experience-sampling survey (*N*_participants_ = 46, *N*_responses_ = 1112), the participants rated their well-being and level of available resources daily for one month. We observed that daily increases in well-being, characterized by higher life satisfaction and lower levels of stress and boredom, were positively associated with more social communication and being outdoors. In summary, the findings support the resource and demand framework, which states that people with access to resources can better cope with the demands of restrictive policies. Implications for policies and interventions to improve well-being are discussed.

## Introduction

The novel coronavirus disease SARS-CoV-2 (COVID-19) outbreak began in December 2019. Humanity responded by developing medication, vaccination, and attempting to stop the spread of the virus and the overburdening of health services through a series of restrictive measures (e.g., "flatten the curve" action). Governmental restrictions included closing public facilities, educational institutions, and borders, enforcing physical distancing, and private quarantine.

These measures were often changing; they differed across countries and sometimes within the same country for different regions. The COVID-19 Stringency Index developed in Oxford provides a composite measure of governmental responses (e.g., school closure, travel bans) and demonstrates the difference in policies across countries and time [1]. Combined with other datasets [e.g., 2], such as the number of confirmed cases or deaths, the efficiency of government responses can be analyzed.

It’s generally agreed that the restrictive measures, such as a lockdown, negatively impacted the economy, e.g., service businesses such as traveling, recreational activities, and restaurants and cultural businesses such as concerts and theater performances. Economic consequences can be measured and tracked. For example, the GDP of Germany decreased by five percent in 2020 [3].

At the same time, behavioral scientists have tried to understand how restrictive measures affected people’s well-being. Wang et al. [4] showed the COVID-19 pandemic and its consequences have increased negative emotions and decreased positive emotions globally. Previous research documented the negative psychological effect of quarantine that may elicit post-traumatic stress symptoms, confusion, and anger [5]. Reappraisal interventions on such emotions worked better during the COVID-19 pandemic when framed positive rather than controlling [4]. Human behavior might change during the lockdown, as there was evidence that unhealthy eating, smoking, and alcohol consumption increased during the lockdown time [6]. Social isolation and loneliness might lead to mental health problems: anxiety, depression, self-harm, and suicide attempts [7]. Comparing measures before and after the beginning of COVID-19, researchers showed an increased level of generalized anxiety in the German population, together with depression symptoms, psychological distress, and COVID-19-related fear [8]. The level of depression and anxiety was also elevated in women during pregnancy at the time of the pandemic [9].

People with preexisting physical or mental health problems were more affected by lockdown and social isolation [7]. For example, individuals with bulimia experienced more negative and less positive emotions after the introduction of lockdown measures. The change in emotions was moderated by the amount of binge eating [10]. Also, individuals with a high level of neuroticism consumed more information related to COVID-19, worried more about the consequences of the crisis, and experienced more negative emotions than the control group [11]. COVID-19 strained the capacity of health services, so people with general disabilities received less rehabilitation than before [12].

Mentally and physically healthy people can also be negatively affected by COVID-19 in terms of their well-being and mental health. In an interview study, 25% of parents stated that their mental health has decreased, and 14% noticed a decrease in their children’s behavioral health since the beginning of the pandemic [13]. Ghosh and colleagues [14] suggested that this negative impact can be explained by the closing of schools, lack of outdoor activity, irregular eating, and changes in sleeping behavior.

Different degrees of impairment during the COVID-19 pandemic were observed between men and women, with women appearing to be more severely affected. Zamarro and colleagues [15] theorized that policies such as school closures and social distancing led women to take more parental duties in the absence of schools and relatives. One indicator for this theory is that in academia, the number of published articles written by women decreased during COVID-19, while the number of publications among men stayed at the same level [16].

The focus of this study is twofold. First, we want to understand how people perceive and get influenced by COVID-19-related restrictive measures. Second, we aim to analyze what influences their well-being daily during the presence of restrictive measures. Findings on factors influencing adaptive capacity and welfare should eventually inform policy to design more effective measures to prevent the spread of the pandemic and ensure people’s well-being.

The response to the COVID-19 pandemic is characterized by an equilibrium between demands, such as wearing masks or limiting the number of contacts, and the resources available to manage them. This equilibrium has been unstable as the regulations have frequently been changing since the pandemic’s beginning.

Cognitive psychology explains how people reflect on their environment; the source of stress is seen as the perceived inability to cope with high demands [17]. Lazarus and Folkman [18] described three main types of stress: challenge, threat, and harm. This typology can be used to distinguish how people perceive the restrictions depending on the amount of coping resources. Whereas some people feel challenged by the need to remain in quarantine, others perceive a threat (e.g., losing a job), and a few undergo harmful consequences (e.g., depression, physical illness). The loss or depletion of resources should cause stress, as described by the conservation of resources theory [19]. Gaining resources, on the other hand, should increase coping abilities and well-being.

For this study, we consider mainly three types of resources: economic (access to financial and material assets, such as physical space), social (stable and meaningful relationships with others), and informational (access to information). Based on viewing changes in COVID-related social regulations (e.g., lockdown) as stress-inducing constraints, we formulated a “resource and demand hypothesis”: We expect that people who have more economic, social, and informational resources will be better adapted to restrictions, experience less stress and have a higher level of well-being.

We present two empirical studies. In the first study, we evaluated in a cross-sectional online survey how the socio-economic characteristics, the resources, and their relative change during COVID-19 were associated with stress elicited by restrictions and with the support of restrictive policies. We found that financial losses due to COVID-19, children in the household, and restrictive measures intensity were associated with higher stress. Lower support for restrictive measures was observed among those who experienced financial losses due to COVID-19, had more children in the household, accessed COVID-19-related information in the media less frequently, and did not self-isolate. In the second study, we analyzed the dynamics of everyday experience to investigate whether the change in the subjective sufficiency of resources was related to well-being. We found that daily increases in well-being, indicated by higher life satisfaction and lower levels of stress and boredom, were positively associated with more social contacts and spending time outdoors.

## Study 1

### Hypotheses

Study 1 focused on how access to different types of resources (economical, socio-emotional, and informational) is associated with 1) how much people are stressed by restrictive measures and 2) how much people support these measures.

Previous research has shown how different resources help people cope with stressful situations. For example, it has been found that social support helps maintain resilience in the face of adversity [20], and a vast network of social ties and personal support helps people cope with high levels of stress [21]. Another study found that people who felt better informed about COVID-19 felt less psychological distress [8]. Therefore, we hypothesized that people with fewer resources should feel more stressed by COVID-19 restrictions.

The amount of stress from the restrictions may be related to attitudes toward the authorities that imposed the restrictions. Previous research has shown that trust in government is tied to people’s satisfaction with public services. High levels of trust in an institution tend to extend to other institutions, while dissatisfaction with a policy may lower support for other policies [22]. Based on these findings, we expected that people who feel more stressed by restrictions would support institutions that imposed restrictions less than people who feel less stressed.

If both hypotheses are valid, i.e., people with fewer resources are more stressed, and more stress is associated with less support, we should also observe that people with fewer resources are less supportive of restrictive policies.

### Method

#### Participants

Six hundred eighty-five participants completed the survey between October 2020 and December 2021 (see Table 1 and Table A1 in Appendix A of S1 File). Mean reported age of participants was 45.7 years (range 18–70, *SD* = 10.9).

**Table 1:** Reported demographic information in Study 1.

	N	%
Gender		
Female	435	63
Male	244	36
Not known	6	1
Having children		
Yes	272	40
No	407	59
Not known	6	1
Employed		
Yes	574	84
No	102	15
Not known	9	1
Education level		
Low	72	10
Middle	353	52
High	254	37
Not known	6	1
Total	685	100

#### Design

Using the Internet to conduct scientific research worldwide has become a successful route to more generalizable results [e.g., 23]. Study 1 was thus conducted as a one-time online survey. The survey was programmed in lab.js [24] and hosted on the online data collection platform Open Lab [25]. The invitation link to the “CoroNOW” survey was placed at the end of the German version of the WageIndicator Survey of Living and Working in Coronavirus Times 2020–2021 [26]. By distributing the link to our survey inside of the WageIndicator Survey, we connected the datasets from both surveys. The WageIndicator survey was used to collect data on the effect of COVID-19 on jobs (e.g., change of workload) and lives of people (e.g., home situation). Our study focused on the number of resources, level of stress, and support of different restrictive measures.

#### Measures

*WageIndicator survey*. In the WageIndicator survey, participants were asked various questions about their living and working conditions [26]. We used the following variables in our analysis: socio-demographic (gender, age) and employment status, education level, number of rooms and children in the household. Education level was measured with the question “What is the highest level of education you have attained?” and response options according to the International Standard Classification of Education 1997 (ISCED-97), which ranges from 0 (No education), 30 (Upper secondary education), 50 (First stage of tertiary education) to 61 (Second stage of tertiary education, leading to an advanced research qualification). For the analysis, we used the recoded variable, which was provided by the WageIndicator survey, that divided the ISCED levels into three groups (0–29 = low, 30–49 = medium, 50–61 = high).

*CoroNow survey*. The survey asked about income level, financial loss, pets in the household, media consumption, isolation, and frustration level (see Table A1 in S1 File for questions). The income level was assessed with a question about the annual income. The financial loss was measured by asking the participants to indicate whether they lost money relative to their monthly income due to COVID-19. The participants were also asked if they had a pet, and if yes, what kind. Media consumption was measured by asking about the frequency of media consumption related to COVID-19. The survey also asked the participants how frustrated they felt with their current situation and whether they had ever self-isolated for at least seven days. According to the National Health Service of the UK, self-isolation was defined as not leaving home if one has symptoms of coronavirus (COVID-19) or lives with someone who does. Self-isolation means not leaving home for any reason other than to exercise once a day, not going out to buy food or collect medicine (but ordering online or asking neighbors and friends to do grocery shopping and pharmacy visits), and not having visitors at home.

To assess their stress level, the participants were asked to what extent various policies in their region constrained them personally. Answers were given on a visual analog scale [27] between “not at all” and “very much”, resulting in values between 0 and 100 inclusive. The support for governmental restrictions was measured with the statement “I agree with the following measures to contain the spread of coronavirus” and the visual analog scale between “Completely disagree” and “Strongly agree”.

The eight policies were similar for both stress and support questions. The order of the items was randomized for each participant.

1. a shutdown of shops and malls
2. closed borders
3. complete lockdown (only shopping and work allowed)
4. missing childcare
5. duty to wear masks
6. social isolation (no meet-ups with more than a fixed number of people)
7. a shutdown of cultural institutions
8. social distancing (the preset number of meters apart)

#### Analysis

The data pre-processing and analysis were performed using R [28]. Descriptive statistics for measured variables are displayed in Table A1 in Appendix A of S1 File. We created the stress and support scores by averaging answers to eight restrictive policies. Test reliability of both scales with Cronbach’s alpha was used as an indication of internal consistency. We used multiple regression models to test whether socioeconomic and personal variables predicted the stress and support scores in the whole sample and its subgroups. Pearson correlations were computed for correlation analysis with *α* = 0.05 (two-sided tests) as the critical significance level. All reported effects are correlative, so they do not imply causality; rather, we interpret the models in terms of the independent variables that explain the stress and support score variance.

#### Ethics statement

Electronic informed consent was obtained before starting the survey and fully complied with IRB regulations at the University of Konstanz. Participation was voluntary and anonymous. Participants could interrupt the survey at any time at no cost. The survey consisted of socio-demographic questions and self-report measures, such as attitudes toward COVID-19 restrictive measures.

### Results

#### Stress and support scales

**Fig 1:**
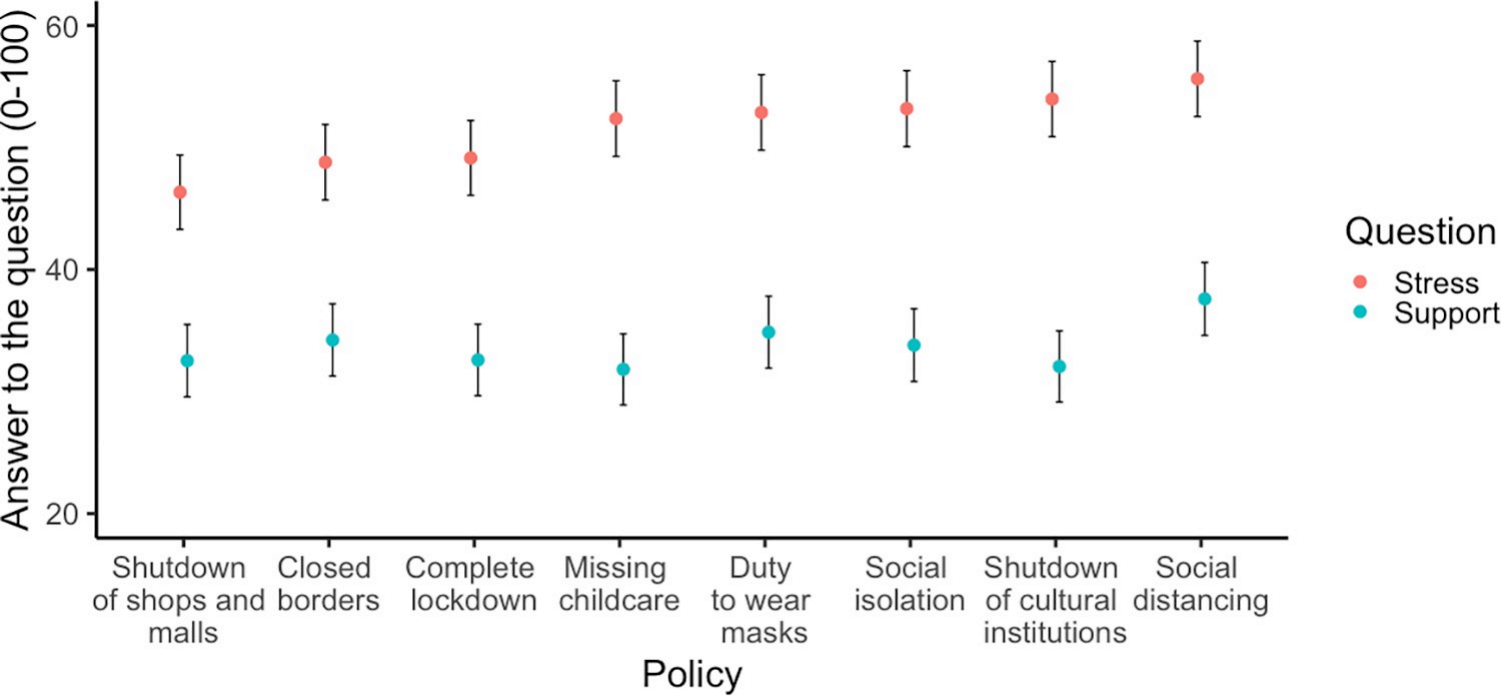
The amount of stress and support of different COVID-19-related policies. Error bars indicate 95% CIs. The answers to the questions about the level of stress and support for each policy were recorded on a scale between 0 and 100. A higher score indicates higher stress levels (in red) or stronger support for restrictive policies (in blue). The policies are ordered by the level of stress.

The eight restrictive measures elicited different stress levels, ranging from a less demanding policy of closing shops and malls to more demanding measures to control social distance (Fig 1). To analyze whether the attitudes toward different restrictive measures are homogeneous at the individual level and can be summarized into an average score, we calculated the intercorrelation between the different policies for the stress and support questions. For the stress score, intercorrelation was high, as indicated by Cronbach’s alpha = 0.81, 95% CI [0.79, 0.83]. For the support score, intercorrelation was very high, Cronbach’s alpha = 0.90, 95% CI = [0.88, 0.91]. In further analyses, the mean scores, indicating the overall stress and support level for each participant, were used.

To assess the external validity of the stress measure, we computed the correlations of the stress score with frustration and life satisfaction measures. The stress score was positively correlated with frustration, *r* = 0.45, *p* < .001; and negatively correlated with life satisfaction, *r* = -0.26, *p* < .001.

#### Hypothesis 1: People with fewer resources are expected to feel more constrained by the restrictions

**Fig 2:**
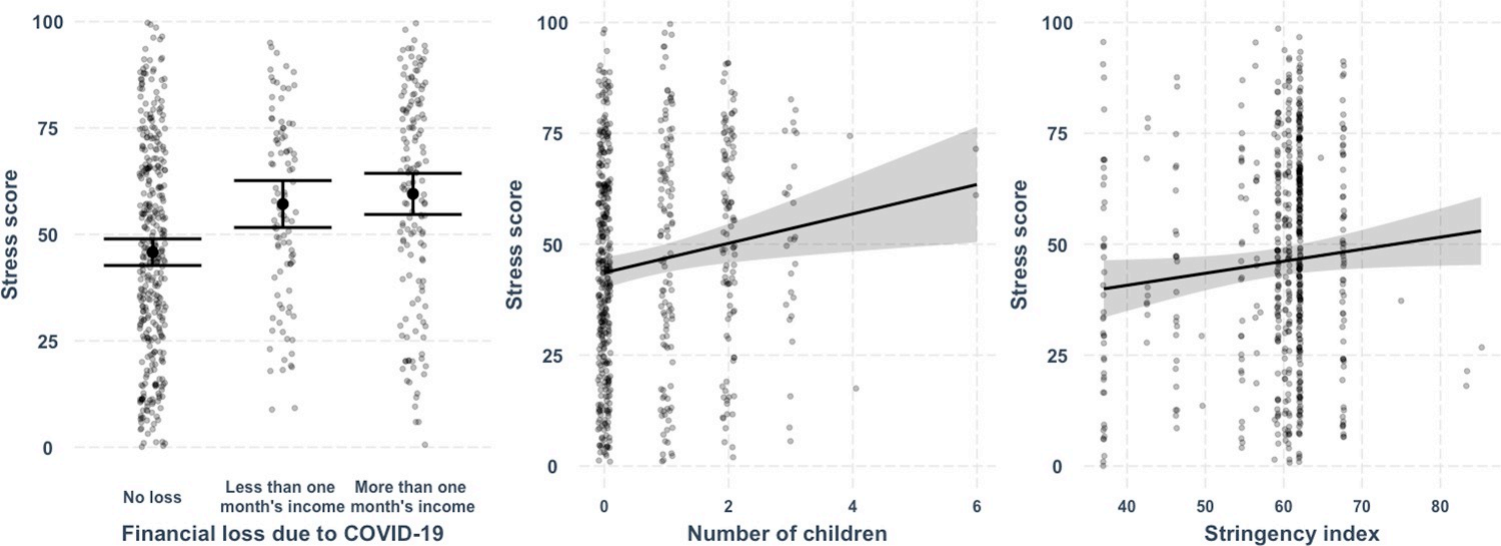
Predicted values of the stress score for different values of financial loss, number of children, and stringency index. 95% confidence intervals are shown for predicted values where all other predictors are held constant, i.e., their mean values are used. Grey dots display the predicted data with the effects of all other predictors accounted for.

The results of the multiple regression model indicate that the predictors explain 10% of the variance in the stress score, *R*^*2*^ = 0.12, *adj*. *R*^*2*^ = 0.10, *F* (14, 615) = 5.75, *p* < .001 (see Table 2). The stress was lower for the participants who did not experience financial loss due to COVID-19, *M* = 46.11, *SD* = 26.46, in comparison to the participants who lost less than one month’s income, *M* = 58.27, *SD* = 23.92, *b* = 11.31, *SE* = 2.96, *p* < .001, and the participants who lost more than one month’s income, *M* = 60.53, *SD* = 27.16, *b* = 13.70, *SE* = 2.58, *p* < .001. The number of children was related to higher stress by restrictive measures, *M*_*no children*_ = 48.34, *SD*_*no children*_ = 25.95, *M*_*one child*_ = 54.24, *SD*_*one child*_ = 29.80, *M*_*two children*_ = 57.52, *SD*_*two children*_ = 27.10, *b* = 3.31, *SE* = 1.21, *p* = .006. The severity of COVID-19 restrictions, as measured by the stringency index, was positively associated with stress, i.e., the higher the stringency index, the more stress participants experienced, *b* = 0.27, *SE* = 0.13, *p* = .043. The average stress score when the stringency index was at the median (*Med* = 60.65) was *M* = 52.52, *SD* = 27.79. The marginal effects of the predictors on the stress score are shown in Fig 2.

**Table 2:** Regression coefficients for Model 1 and Model 2.

	Model 1—Stress			Model 2—Support		
*Predictors*	*Estimates*	*CI*	*p*	*Estimates*	*CI*	*p*
(Intercept)	32.97	12.05 – 53.89	0.002	29.08	6.81 – 51.35	0.011
Male (vs. Female)	3.00	-1.50 – 7.51	0.19	-8.12	-12.92 – -3.32	0.001
Age	-0.13	-0.33 – 0.06	0.18	-0.08	-0.29 – 0.12	0.42
Education level	1.19	-2.20 – 4.58	0.49	1.73	-1.88 – 5.34	0.35
Employment	3.38	-2.54 – 9.31	0.26	-5.90	-12.21 – 0.41	0.07
Income level	0.77	-0.57 – 2.12	0.26	-0.82	-2.25 – 0.61	0.26
Rooms per person	-1.39	-4.02 – 1.25	0.30	2.21	-0.59 – 5.02	0.12
Financial loss (less than one month income)	11.31	5.50 – 17.11	<0.001	-11.05	-17.23 – -4.87	<0.001
Financial loss (more than one month income)	13.70	8.62 – 18.77	<0.001	-18.18	-23.58 – -12.78	<0.001
Number of children	3.31	0.93 – 5.69	0.006	-4.21	-6.74 – -1.67	0.001
Home pet	0.29	-3.93 – 4.50	0.89	-2.43	-6.92 – 2.05	0.29
Information access	-1.67	-3.66 – 0.32	0.10	3.06	0.93 – 5.18	0.005
Self-isolation	-5.48	-11.18 – 0.22	0.06	9.12	3.06 – 15.19	0.003
Stringency index	0.27	0.01 – 0.53	0.043	0.19	-0.09 – 0.47	0.19
New COVID-19 cases (in thousands)	0.19	-0.10 – 0.48	0.20	-0.39	-0.69 – -0.08	0.014
Observations	630			630		
R^2^ / R^2^ adjusted	0.12 / 0.10			0.17 / 0.15		

To investigate how predictors were related to stress by a specific restrictive policy, we have repeated the analysis for each policy. The significance level of coefficients was set to *α* = .006 to correct for multiple comparisons. Losing more than one month’s income compared with no loss showed a higher stress score for each of the policies except complete lockdown. The number of children and the stringency index did not have significant associations with the stress caused by a specific policy.

### Hypothesis 2: People with fewer resources should show a lower level of support

**Fig 3:**
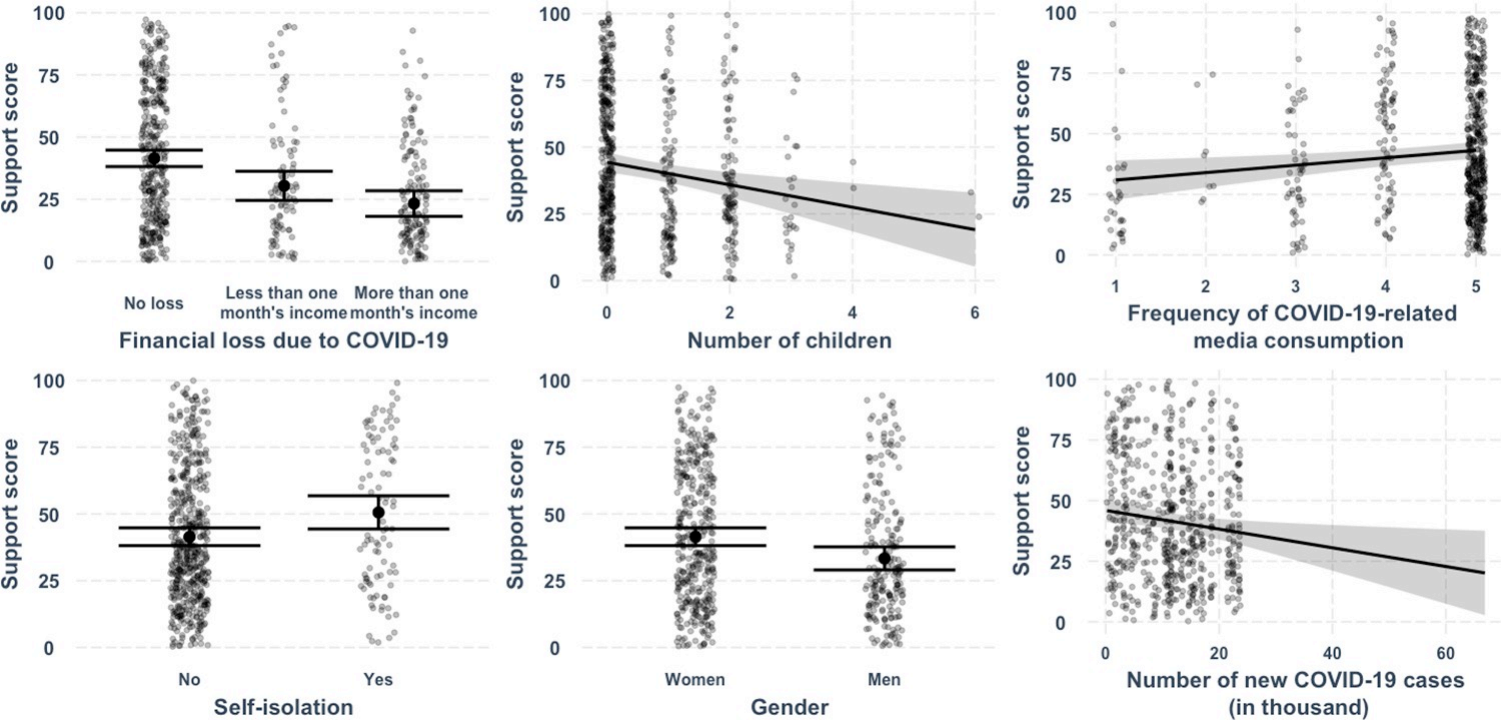
Predicted values of the support score for different values of financial loss, number of children, media consumption, self-isolation, new COVID-19 cases, and gender. 95% confidence intervals are shown for predicted values where all other predictors are held constant, i.e., their mean values are used. Grey dots display the predicted data with the effects of all other predictors accounted for.

The results of the multiple regression model indicate that the predictors explain 15% of the variance in the support score, *R*^*2*^ = 0.17, *adj*. *R*^*2*^ = 0.15, *F* (14, 615) = 9.20, *p* < .001. The support was higher for the participants who did not experience financial loss due to COVID-19, *M* = 40.28, *SD* = 31.09, in comparison to the participants who lost less than one month’s income, *M* = 28.17, *SD* = 27.55, *b* = -11.05, *SE* = 3.15, *p* < .001, and the participants who lost more than one month’s income, *M* = 21.13, *SD* = 23.11, *b* = -18.18, *SE* = 2.75, *p* < .001. The support for restrictive policies was higher for those participants who accessed COVID-19-related information more frequently, *M*_*less than once a week*_ = 17.24, *SD*_*less than once a week*_ = 22.44, *M*_*several* times a day_ = 33.40, *SD*_*several* times a day_ = 30.30, *b* = 3.06, *SE* = 1.08, *p* = .005. Individuals who self-isolated supported the restrictions more than individuals who did not self-isolate, *M* = 43.53, *SD* = 33.63 vs. *M* = 31.92, *SD* = 28.92, *b* = 9.12, *SE* = 3.09, *p* = .003. Individuals with children were less supportive of restrictions, *M*_*no children*_ = 37.92, *SD*_*no children*_ = 30.51, *M*_*one child*_ = 28.67, *SD*_*one child*_ = 29.07, *M*_*two children*_ = 27.37, *SD*_*two children*_ = 28.07, *b* = -4.21, *SE* = 1.29, *p* = .001. Men were less supportive of restrictions than women, *M* = 26.98, *SD* = 28.21 vs. *M* = 37.40, *SD* = 30.23, *b* = -8.12, *SE* = 2.44, *p* = .001. An increase in the number of new COVID-19 cases was associated with less support, *b* = -0.39, *SE* = 0.16, *p* = .014. The average support score when the number of new COVID-19 cases was at the median (in thousands, *Med* = 11.52) was *M* = 40.44, *SD* = 30.32. The marginal effects of the predictors on the stress score are shown in Fig 3.

To investigate how predictors were related to the support for a specific restrictive policy, we have repeated the analysis for each policy. The significance level of coefficients was set to *α* = .006 to correct for multiple comparisons. Losing more than one month’s income compared to no losing showed less support for each policy. The significant negative association of the number of children with support was present only for the shutdown of shops and malls, complete lockdown, social isolation, and missing childcare policies. More frequent COVID-19-related media consumption was positively associated with higher support for the shutdown of shops and malls and complete lockdown policies. Self-isolation was positively related to higher support for the closed-borders policy. Men supported the duty to wear masks, social isolation, and social distancing policies less than women. The number of new COVID-19 cases was positively associated with reduced support for the social distancing policy.

#### Hypothesis 3: People who feel more constrained by the restrictions are expected to support them less than people who do not feel constrained

**Fig 4:**
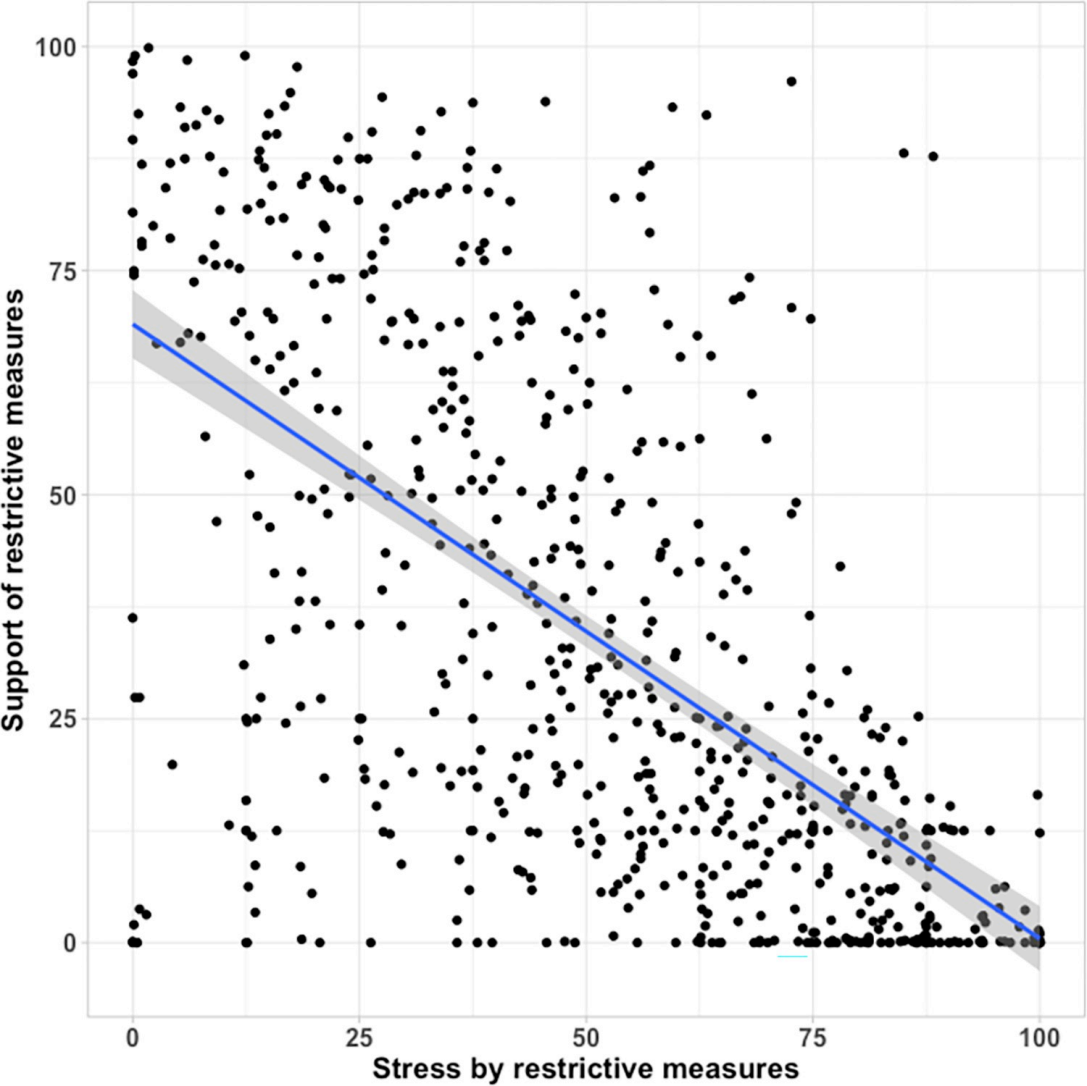
Stress and support scores.

Participants who felt more stressed by the restrictions supported them less than participants who felt less stressed (Fig 4), as indicated by the negative correlation between the stress and support scores, *r* = - 0.62, *p* < .001.

#### Exploratory analysis

In the following section, we report an analysis of interactions between different factors in order to better understand how individuals in different groups (e.g., individuals who experienced financial losses due to COVID-19 and individuals who did not) perceived restrictive measures.

*Financial loss*. We have found that the participants with higher financial loss reported higher stress levels and lower support levels for restrictive measures. We explored the differences between the groups with different levels of financial loss with respect to other variables. The groups did not differ significantly in age, gender, income, education, number of rooms per person in the household, number of children, ownership of a pet, and frequency of consumption of COVID-19-related information (*p*s > .05, see Table A2 in Appendix A of S1 File). In the group that lost more than one month’s income, there were more unemployed individuals, *N*_*no loss*_ = 42 (10%) vs. *N*_*less*_ = 13 (13%) vs. *N*_*more*_ = 45 (28%), *X*^*2*^(2, *n* = 668) = 28.36, *p* < .001. Experiencing financial loss was associated with a higher level of frustration with the current situation, *M*_*no loss*_ = 61.89, *SD*_*no_loss*_ = 36.53 vs. *M*_*less*_ = 68.12, *SD*_*less*_ = 34.26 vs. *M*_*more*_ = 85.25, *SD*_*more*_ = 25.73, *F*(2, 674) = 27.67, *p* < .001, and a lower level of life satisfaction, *M*_*no loss*_ = 5.55, *SD*_*no_loss*_ = 2.61 vs. *M*_*less*_ = 5.36, *SD*_*less*_ = 2.43 vs. *M*_*more*_ = 4.16, *SD*_*more*_ = 2.66, *F*(2, 667) = 16.84, *p* < .001.

**Fig 5:**
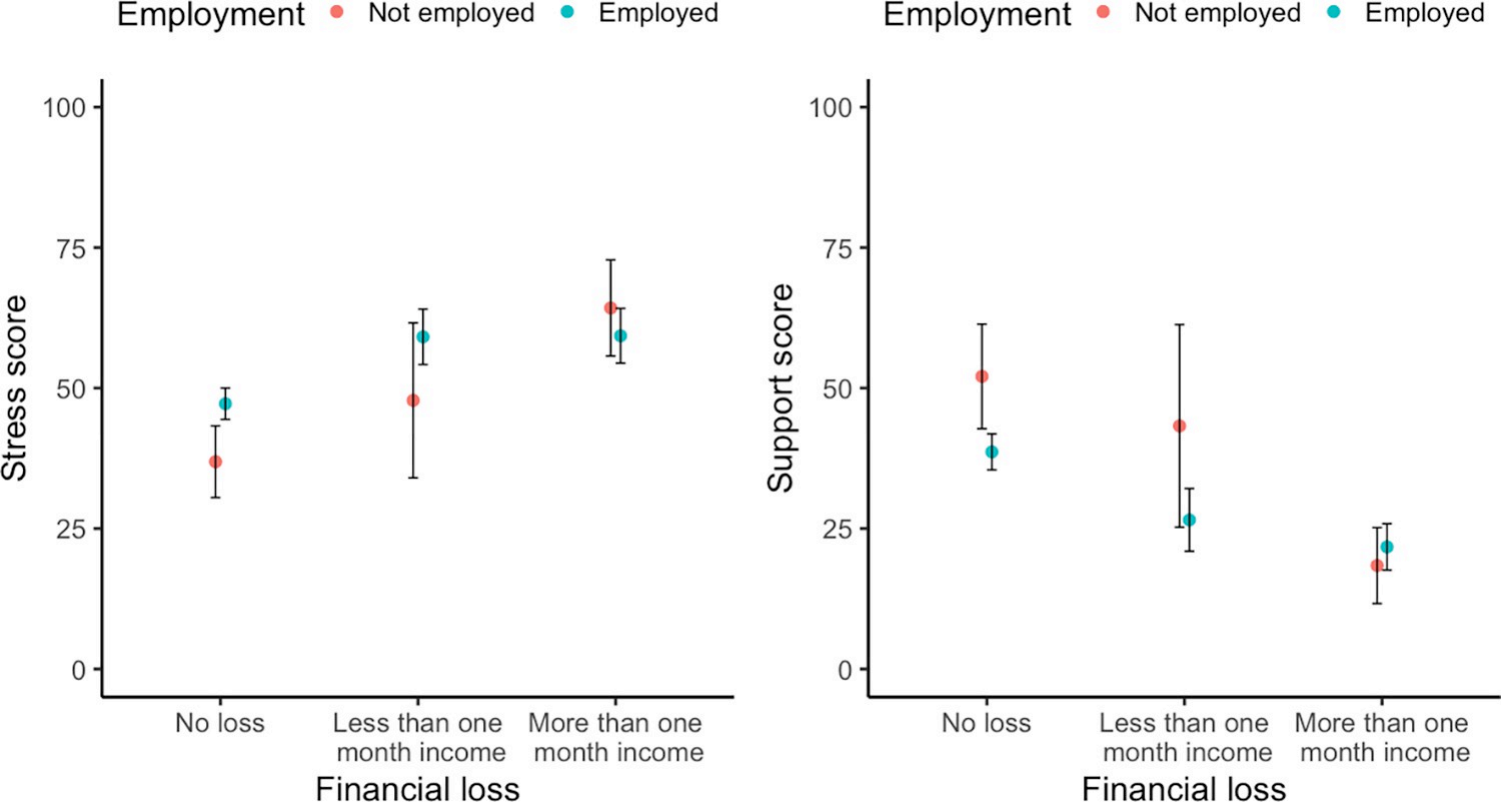
Stress and support scores by employment and amount of financial loss due to COVID-19.

Stress and support levels differed between the financial loss and no loss groups depending on whether they were employed or not, as confirmed by a significant interaction between financial loss of more than one month’s income and employment for stress, *b* = -15.26, *SE* = 6.26, *p* = 0.015, and support scores, *b* = 16.76, *SE* = 6.82, *p* = 0.014 (see Table A3 in Appendix A of S1 File for regression coefficients). While in the no-loss group, employed individuals experienced more stress than non-employed individuals, *M* = 47.21, *SD* = 26.93, vs. *M* = 36.90, *SD* = 20.48, in the group that lost more than one month’s income, employed individuals reported less stress than non-employed individuals, *M* = 59.31, *SD* = 26.52, vs. *M* = 64.26, *SD* = 28.47. A similar pattern was observed for the support score. In the no-loss group, employed individuals were less supportive of the restrictive policies than non-employed individuals, *M* = 38.64, *SD* = 30.91 vs. *M* = 52.08, *SD* = 29.87. In the group that lost more than one month’s income, employed individuals were more supportive of the restrictions than non-employed individuals, *M* = 21.73, *SD* = 22.46 vs. *M* = 18.41, *SD* = 22.49 (Fig 5).

*Stringency index*. Because the intensity of COVID-19 restrictive measures (as measured by the stringency index) was positively related to the level of stress they induced, we further examined the interaction between the stringency index and other variables, i.e., how various factors were associated with stress and support for restrictive measures during periods when the stringency index was low versus when it was high. We tested extended versions of Model 1 and Model 2 where the interaction between the COVID-19 stringency index and the financial loss, the number of children, media consumption, self-isolation, gender, or the number of new COVID-19 cases were included. None of the interactions between the stringency index and the other variables were statistically significant (*p*s > .05).

To further investigate the possible non-linear interactions between the stringency index and other variables, we repeated the regression analysis for the two groups: the group in which the stringency index was below or equal to the 25^th^ percentile (“low stringency index”, *n* = 232) and the group in which the stringency was above or equal to the 75^th^ percentile (“high stringency index”, *n* = 334). The groups did not differ significantly in age, gender, employment, income, financial loss, number of rooms per person in the household, ownership of a pet, and number of children (*p*s > .05). The high stringency index group had a higher level of media consumption than the low stringency index group, *M*_*low*_ = 4.22, *SD*_*low*_ = 1.20 vs. *M*_*high*_ = 4.55, *SD*_*high*_ = 0.96, *t* (560) = -3.56, *p* < .001.

**Fig 6:**
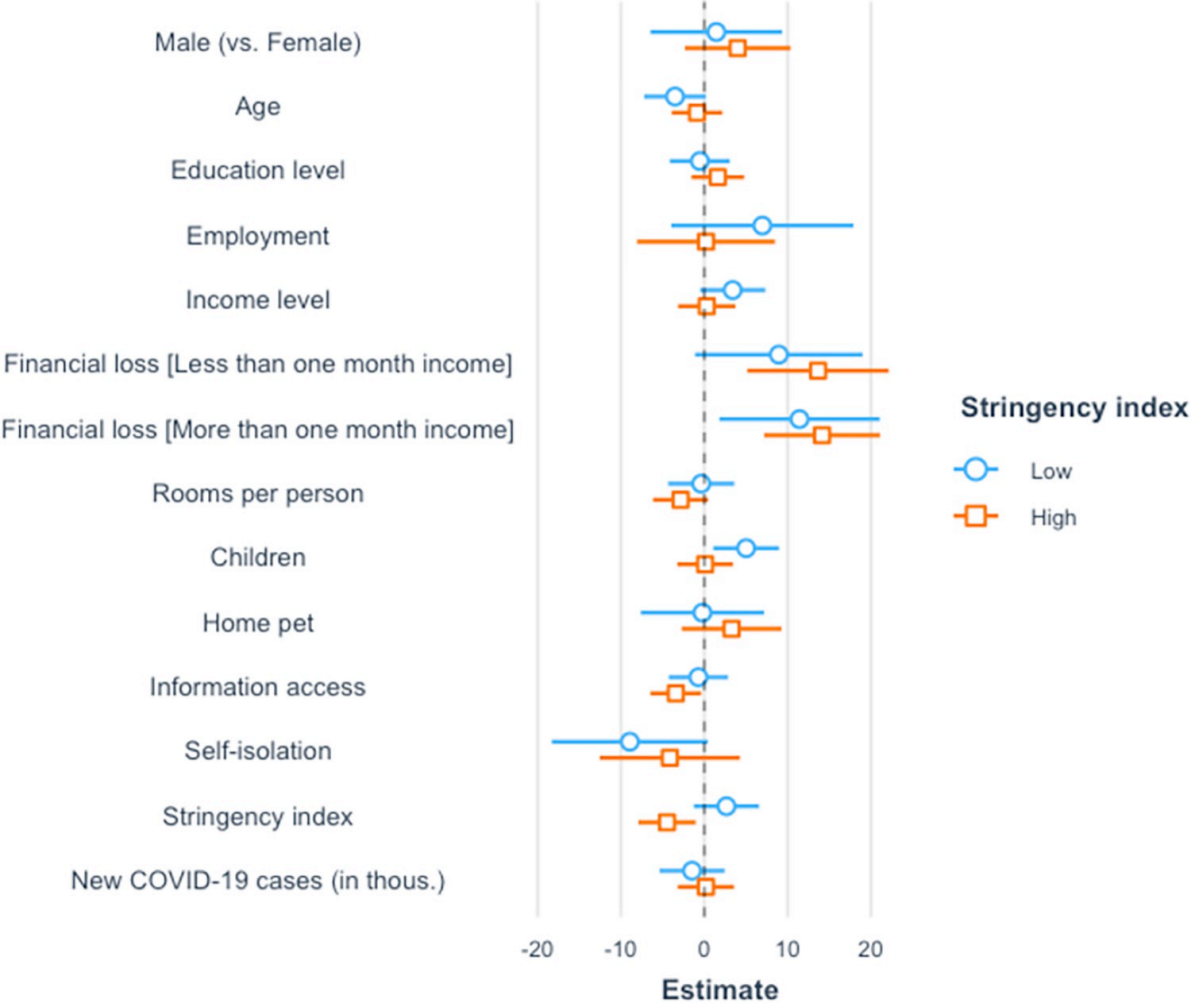
Standardized regression coefficients for stress in groups with different level of stringency index. Standardized regression coefficients with 95% CIs are shown. The confidence interval of the effect estimate should not overlap with zero for the effect to be considered statistically significant.

With regard to the stress elicited by restrictive measures (Fig 6), loss of more than one month’s income was associated with higher stress in both low and high stringency index groups, *b* = 11.42, *SE* = 4.88, *p* = .02, and *b* = 14.13, *SE* = 3.54, *p* < .001. The media consumption and the stringency index were negatively associated with stress only in the high stringency index group, *b* = -3.51, *SE* = 1.59, *p* = .028, and *b* = -1.43, *SE* = 0.56, *p* = .011. In contrast, the positive association between the number of children and stress was present only in the low stringency index group, *b* = 5.35, *SE* = 2.14, *p* = 0.013.

**Fig 7:**
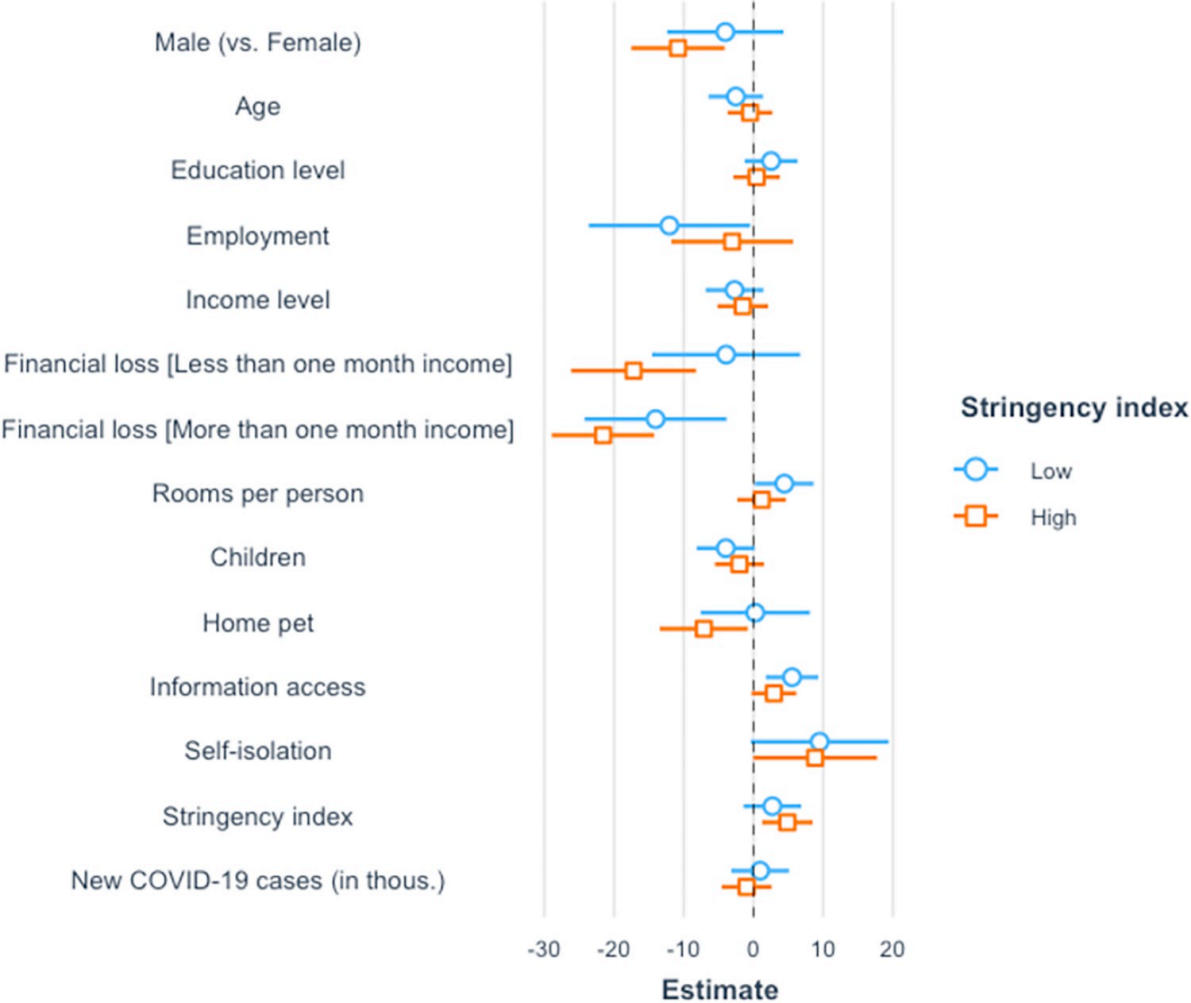
Standardized regression coefficients for support of restrictive measures in groups with different level of stringency index. Standardized regression coefficients with 95% CIs are shown. The confidence interval of the effect estimate should not overlap with zero for the effect to be considered statistically significant.

Regarding the support for restrictive measures (Fig 7), losing more than one month’s income was associated with lower support in both low and high stringency index groups, *b* = -14.04, *SE* = 5.16, *p* = .007, and *b* = -21.58, *SE* = 3.73, *p* < .001. The effects of other variables differed for low and high levels of restrictive measures. When the stringency index was low, being employed was associated with less support, *b* = -12.05, *SE* = 5.87, *p* = .041, while more rooms per person and more frequent media consumption increased support, *b* = 5.05, *SE* = 2.43, *p* = .039, and *b* = 4.82, *SE* = 1.67, *p* = .004. When the stringency index was high, on the other hand, losing less than one month’s income reduced support, *b* = -17.20, *SE* = 4.55, *p* < .001. Also, male gender and pet ownership were negatively related to support, *b* = -10.83, *SE* = 3.41, *p* = .002, and *b* = -7.12, *SE* = 3.21, *p* = .027. However, the increase in stringency index was associated with the increased support for restrictive measures, *b* = 1.55, *SE* = 0.59, *p* = .009.

We further explored which kind of pet was associated with less support for restrictive policies during the period when the stringency index was high. To analyze that, we entered dummy variables in the multiple linear regression model for owning a cat (31% of participants had at least one cat), a dog (20%), fish (4%), a small pet such as a hamster, guinea pig, or mouse (5%), or another type of pet (5%). Only owning a cat was negatively related to the support level, *b* = -7.04, *SE* = 3.48, *p* = 0.044. The effects of other types of pets were not significant, *p*s > 0.05.

Regarding the specific type of restrictive policy, owning a cat did not have significant associations given that the significance level of coefficients was set up to α = .006 to correct for multiple comparisons. Cat owners did not differ significantly from others in age, gender, education, employment, income, financial loss, number of rooms per person in the household, and frequency of consumption of COVID-19-related information. However, cat owners had more children than the others, *M*_*cat*_ = 0.88, *SD*_*cat*_ = 1.08 vs. *M*_*no cat*_ = 0.52, *SD*_*no cat*_ = 0.83, *t* (330) = -3.27, *p* = .001.

*Self-isolation*. We used a multiple logistic regression analysis to examine which factors were associated with self-isolation. The decision to self-isolate was positively related to losing more than one month’s income, *b* = 2.04, *SE* = 0.55, *p* = 0.008, media consumption, *b* = 1.40, *SE* = 0.19, *p* = 0.014, and new COVID-19 cases, *b* = 0.96, *SE* = 0.02, *p* = 0.037.

*Life satisfaction*. To answer the question of how restrictive measures did affect well-being, we conducted a multiple regression model analysis. The model’s predictors explain 15% of the variance in life satisfaction, *R*^*2*^ = 0.17, *adj*. *R*^*2*^ = 0.15, *F* (16, 613) = 7.65, *p* < .001 (see Table 3). Higher life satisfaction was associated with male gender, age, education level, employment, and number of children. People who lost more than one month’s income were less satisfied with their life. The feeling of stress by restrictive measures was negatively associated with life satisfaction, and support for restrictive measures, on the other hand, was positively related to life satisfaction.

**Table 3:** Regression coefficients for life satisfaction.

	Life satisfaction		
*Predictors*	*Estimates*	*CI*	*p*
(Intercept)	3.06	1.01 – 5.10	0.003
Male (vs. Female)	0.58	0.15 – 1.02	0.008
Age	0.02	0.00 – 0.04	0.033
Education level	0.45	0.13 – 0.78	0.006
Employment	0.95	0.38 – 1.51	0.001
Income level	-0.05	-0.18 – 0.08	0.47
Rooms per person	0.14	-0.11 – 0.39	0.27
Financial loss (less than one month income)	0.26	-0.30 – 0.81	0.70
Financial loss (more than one month income)	-0.90	-1.40 – -0.40	<0.001
Number of children	0.24	0.01 – 0.47	0.041
Home pet	-0.15	-0.55 – 0.25	0.46
Information access	-0.05	-0.24 – 0.15	0.64
Self-isolation	0.02	-0.52 – 0.57	0.94
Stress by restrictive measures	-0.02	-0.03 – -0.01	<0.001
Support for restrictive measures	0.01	0.00 – 0.02	0.013
Stringency index	0.00	-0.02 – 0.03	0.89
New COVID-19 cases (in thousands)	-0.02	-0.05 – 0.00	0.08
Observations	630		
R^2^ / R^2^ adjusted	0.17 / 0.15		

### Discussion

In Study 1, we examined factors related to stress and support for restrictive measures, such as a lockdown or wearing masks policy. We found that financial loss, more children in the household, and more intense restrictive measures were associated with higher stress levels. Concerning the support for the restrictive policies, individuals who felt more stressed by the restrictions showed less support. Financial losses and the number of children also played a role here–individuals with losses and more children demonstrated less support for restrictions. In addition, several other variables were related to the level of support–men (compared with women), individuals who did not self-isolate, and individuals who accessed less COVID-19-related information were less supportive of restrictive measures. The number of new COVID-19 cases was also negatively related to the level of support. We discuss each of these factors in more detail below.

#### Financial loss and employment

Individuals who experienced financial losses generally were more stressed and less supportive of most restrictive measures, which was also consistent with higher levels of frustration and lower life satisfaction. Loss of resources is a cause of stress, which is predicted by the conservation of resources theory [19] and supported by previous research [20]. Importantly, our results indicate that financial loss can be more relevant than income level in predicting stress and support for restrictive measures. Income level did not appear to be a significant factor in our study. This is in line with the research by Beland and colleagues [29]. They found that employment status and work arrangements during COVID-19 (such as remote working) were not related to self-reported levels of family stress, but the inability to meet financial obligations increased reported family stress and domestic violence. The income level, however, might play a role in other COVID-19-related decisions, such as the vaccination decision. For instance, Peretti-Watel and colleagues [30] found that people with a low income were more reluctant to get vaccinated.

In our study, individuals who had lost more than one month’s income were more often unemployed than others. At the same time, combination of unemployment and financial loss of more than one month’s income was associated with the highest level of stress and the lowest level of support for restrictive measures. It is possible that unemployment was related to COVID-19, so the loss of a job exacerbated the loss of income. About 1.09% of the population in Germany is estimated to have lost their job due to COVID-19 [31]. Thus, loss of job and income could be indicators of vulnerability. As discussed in previous research, vulnerable groups are not only defined by age, illness, and homelessness, but may also represent various socioeconomic groups that have difficulty coping financially with the crisis [32].

Employment itself did not guarantee a lower stress from restrictive measures, as shown by the interaction of employment and financial loss in our dataset. In the group without financial loss, employed individuals were more stressed by restrictive measures than were non-employed individuals, which could be related to the extent to which the restrictions affected their work and challenged them to adapt to a new situation (e.g., working remotely, wearing a mask at work).

#### Children

Having children was related to higher well-being, as individuals with children showed higher life satisfaction. At the same time, having children became associated with more stress by restrictive measures and less support for restrictions during COVID-19. These results coincide with other research showing that parental stress increased during the pandemic [33]. In particular, Calvano and colleagues [33] reported that more than 50% of parents were stressed by social distancing, closure of schools and childcare facilities. We found similar patterns, as people with children in our study were less supportive of social isolation and childcare policies. Surprisingly, the association between stress and the number of children was not present when the stringency index was high. Perhaps, when the restrictive measures were intense, such as during the complete lockdown, other factors mitigated the association between children and stress.

#### Stringency index and the number of new COVID-19 cases

More stringent restrictive measures were associated with higher stress levels but were not related to policy support. In contrast, the increasing number of new COVID-19 cases were not related to the stress caused by restrictive measures but reduced support for them. It can be that when the number of new COVID-19 cases was large, the restrictive measures were considered less effective, which was reflected in a lower level of endorsement.

The different intensity levels of restrictions might represent different challenges for the population, so the stringency index’s effect is not linear. Exploring the differences between the high and low stringency index times, we found that owning a home pet, particularly a cat, was associated with less support for restrictive policies during the high stringency index times. It is generally accepted that pet ownership can bring positive emotions of caring, companionship, and the human-animal bond. During COVID-19, many pet owners reported that their pets gave them emotional comfort and positively impacted their lives [34]. In our study, we could not find support for the benevolent effects of a home pet, neither with respect to the stress by restrictive measures nor life satisfaction. On the contrary, we found that cat owners were less supportive of restrictive regulations than others when the restrictive regulations were relatively intense. Having a cat at home during the complete lockdown could mean difficulties in caring for the animal, such as visiting a veterinarian [35]. Cat owners could feel more restricted by the complete lockdown, which was not the case for dog owners. While owning a dog during the lockdown might have some benefits, such as an excuse to go outside, keeping a cat could not provide this advantage.

Another factor that was relevant only to the specific level of the severity index was household crowding, measured as the number of rooms per person. It was not associated with life satisfaction or stress from restrictive measures. However, when restrictive measures were low, people with more living space were more likely to endorse restrictions. Because people have been spending much more time at home during the lockdown, the size of their living space became more essential for their well-being. This is in line with previous research that has shown that living in an overcrowded household can be associated with higher levels of distress and that stress levels have even increased since the beginning of lockdowns; e.g., in April 2020, 39% of people in overcrowded households in the UK reported feeling distressed compared to 29% in non-overcrowded households [36]. Overcrowding might also be associated with the financial pressure of housing payments, as overcrowded households might seek to share accommodation costs by sharing them with more people.

#### Gender

Men were generally less supportive of COVID-19 restrictive measures than women. In particular, this difference was present across several policies: duty to wear masks, social isolation, and social distancing. Other studies confirm this finding, indicating that women are more likely to perceive COVID-19 as a severe health problem and comply with restrictive measures [37]. As Galasso and colleagues [37] argue, these differences can explain the lower COVID-19 mortality among women and the more efficient response of women-led countries to the pandemic [see also 38]. Women are also less likely than men to endorse COVID-19 conspiracy theories [39].

#### Self-isolation

Individuals who had done self-isolation were more supportive of COVID-19-related restrictions. The individuals who had isolated themselves might be exposed to COVID-19 either by being sick themselves or living with someone who was sick. Additionally, it could be that people who followed COVID-19-related recommendations were more likely to impose self-isolation on themselves. The positive association between media consumption and the decision to self-isolate can indicate that more informed people were more willing to self-isolate but also that people during self-isolation might consume more COVID-19-related information. It has been argued that the ability to isolate depends on people having the space and resources to do so, and lost wages can be the primary reason for not following guidelines [40]. In contrast, we found that losing one month’s income was positively associated with self-isolation, suggesting that losing wages might also be a consequence of self-isolation.

#### Media consumption

The media consumption about COVID-19 can increase trust in governmental actions [41] and compliance with prevention measures [42], but on the other hand, can induce state anxiety when it is excessive [43, 44]. Indeed, in our study, we found that more frequent access to COVID-19-related information was positively associated with support for restrictive policies. Additionally, this effect was more pronounced when the stringency index was high. The reason for the positive correlation might be a causal influence of one variable on the other (higher support increases media consumption or more media consumption increases support) or a third variable that influences both support and the amount of media consumption. One possible mediator between media consumption and support of restrictions could be COVID-19-related fear. Previous research has shown that increased media exposure was associated with depression and COVID-19-specific anxiety in Germany [45]. Media exposure can increase the level of fear, and fear can justify the use of restrictive policies to curb the spread of the virus.

#### Life satisfaction

Socio-demographic and socio-economic characteristics such as male gender, older age, education level, and employment were positively related to life satisfaction. Many previous studies before COVID-19 found that life satisfaction did not depend on gender [46, 47]. Given that COVID-19 affected men and women unequally and often disadvantaged women [15], this may explain why women reported lower levels of life satisfaction in our study. The effects of age, education, and employment on life satisfaction are similar to results from previous studies [46]. More importantly, stress from restrictive measures, but not the stringency index itself, was negatively related to life satisfaction, confirming that psychological well-being is not determined by stressful events but by how individuals perceive and adapt to them.

To summarize the results of Study 1, we found that people’s psychological well-being during COVID-19 was affected by the restrictive measures. Using a cross-sectional survey, we examined the role of demographic and socioeconomic individual differences in response to restrictive measures. Stress and the level of support for restrictive measures were related to socioeconomic factors associated with people’s level of resources, such as financial loss. To complement these findings with people’s perception of their own resources, we conducted a second study that focused on how people perceive the amount of resources available to them. In addition, we wanted to assess the dynamic relationship between resources and well-being: How do changes in the amount of available resources affect well-being? To answer this question, Study 2 was conducted as an experience sampling study with repeated measures of perceived amount of available resources and well-being.

## Study 2

### Hypotheses

Study 1 confirmed that the loss of material resources was related to stress from restrictive measures. To examine how daily resource changes are related to well-being, we conducted Study 2. We used the experience sampling method to collect data over one month. According to the *resource and demand hypothesis*, positive resource change should be associated with better well-being. Specifically, we expect that an increase in material resources (e.g., food), social contacts, and information should reduce stress and negative emotions and improve life satisfaction.

### Method

#### Participants and COVID-19 situation

Forty-six students of the University of Konstanz (38 women, five men, and three unknown) completed the study, which lasted four weeks (see Table A4 in Appendix A of S1 File for summary statistics). The mean age of participants was 21.3 years (range 19–26, *SD* = 1.98). The study period was in March to May, 2021 (not all participants began on the same date). During this period, the stringency index in Germany decreased from 81.48 before the 1st of March to 77.78 from the 1^st^ to the 20^th^ of March to 75 after the 20^th^ of March. The decrease in the stringency index indicates that COVID-19 restrictions were loosening during this period. The number of confirmed COVID-19 cases per day increased from 6,248 on the 1^st^ of March to 11,783 on the 21^st^ of April and then decreased to 1,971 on the 31^st^ of May [48].

#### Design

Participation in the study began with an online onboarding survey similar to the survey from Study 1. Because the participants had not taken the WageIndicator survey, the onboarding survey included questions about country and city of residence, household size (rooms, people), and a positive COVID-19 test in the past. The participants were then asked to complete a daily survey over four weeks. Each day, at a randomly selected time between 9 am and 9 pm, they received a notification via the "Samply research" mobile application with a link to the survey. The order of questions in the daily survey was randomized for each participant and each day.

#### Measures

*Onboarding survey*. The content of the onboarding survey was similar to the "CoroNOW survey" used in Study 1. Because the purpose of Study 2 was to examine daily changes in resource levels and well-being, our analysis focuses on the daily survey results.

*Daily survey*. The daily survey measured life satisfaction, negative emotions, and sufficiency of material, social, and informational resources (see Table 4). All responses were given on a visual analog scale and saved as values from 0 to 100. Life satisfaction was measured with the question "How satisfied are you with your life at the current moment?”, and the end labels were “Not at all” and “Very much satisfied”. Negative emotions included questions about anxiety, worry about significant others, financial concerns, boredom, and stress, and the end labels were “Not at all” and “Very much”. Finally, participants were asked to what extent the amount of material resources, social contacts, and information about COVID-19 is currently sufficient for them. End labels ranged from “Completely insufficient” to “Completely sufficient”. The order of the questions was randomized for each participant and each measurement. We also asked participants whether they were currently outdoors and whether they had been outdoors the previous day.

**Table 4:** Daily survey items.

Item	Question (translated from German)
Life satisfaction	How satisfied are you with your life at the current moment?
Fear	How much do you fear to be infected at the current moment?
Worries about significant others	How worried are you at this moment that your loved ones might be infected?
Economic concern	How concerned are you about your potential economic loss at this moment?
Boredom	How much are you bored at the current moment?
Stress	How stressed do you feel at the current moment?
Material resources	To what extent is your today’s basic supply (e.g. food, water, household goods) sufficient?
Social communication resources	How far is your today’s social communication sufficient for your normal level?
Information resources	To what extent is the level of information about the current situation related to COVID-19 (e.g., guidance from the authorities, news) you receive sufficient?

*Debriefing*. At the end of the study participants received a debriefing, in which we thanked them for participation and provided instructions how to receive participation credits.

#### Procedure

The study participants were recruited via the online recruitment platform Sona at the University of Konstanz. Participation was rewarded with course credit points. The study advertisement indicated that participants needed to have a smartphone with Internet access to participate. Interested participants were invited to take the onboarding survey, which could be completed on desktop computers or laptops. At the end of the onboarding survey, participants were instructed to install the “Samply research” mobile application [available at https://samply.uni-konstanz.de/, 49] and participate in the daily survey there. Participants were asked to join the study immediately after the onboarding survey, and the sampling schedule began the next day and lasted for four weeks. After completing the daily survey, participants received a debriefing the next day, thanking them for their participation and giving them instructions on obtaining their credit points.

#### Analysis

Data preprocessing and analysis were done using R [28]. Descriptive statistics were calculated for measured variables (see Table A4 in Appendix A of S1 File). We applied linear mixed-effects models using the *nlme* package [50] to analyze the effects of resources on well-being. Following recommendations for the longitudinal data analysis [51], we separated between- and within-subjects effects. The model was corrected for autocorrelation errors, and the measurement day was included in the analysis to control for the influence of time. The independent variables were centered on the mean to facilitate the interpretation of the model results. We used random intercepts for each participant and random slopes (days within participants) as random effects. The model estimated the fixed effects of the sufficiency of information, communication, and material resources (both at the between- and within-subject levels) on the well-being score. Other fixed effects were the measurement day, being outdoors at the moment and yesterday, the stringency index, and the number of new COVID-19 cases.

#### Ethics statement

Electronic informed consent was obtained before starting the survey and fully complied with IRB regulations at the University of Konstanz. Participation was voluntary, and participants could interrupt the study at any time. The survey consisted of socio-demographic items and self-report measures.

### Results

#### Well-being score

The well-being score was calculated as the average of the life satisfaction and negative emotion scores (stress, fear, boredom, worries, and financial concern), which were reversed by subtracting from 100. The intercorrelation of items in the well-being score was moderate, as indicated by Cronbach’s alpha = 0.60, 95%CI [0.57, 0.64]. The mean well-being score across participants was 66.20 (range 34.90–89.20, *Med* = 66.90, *SD* = 11.27).

#### The effect of resources on well-being

**Fig 8:**
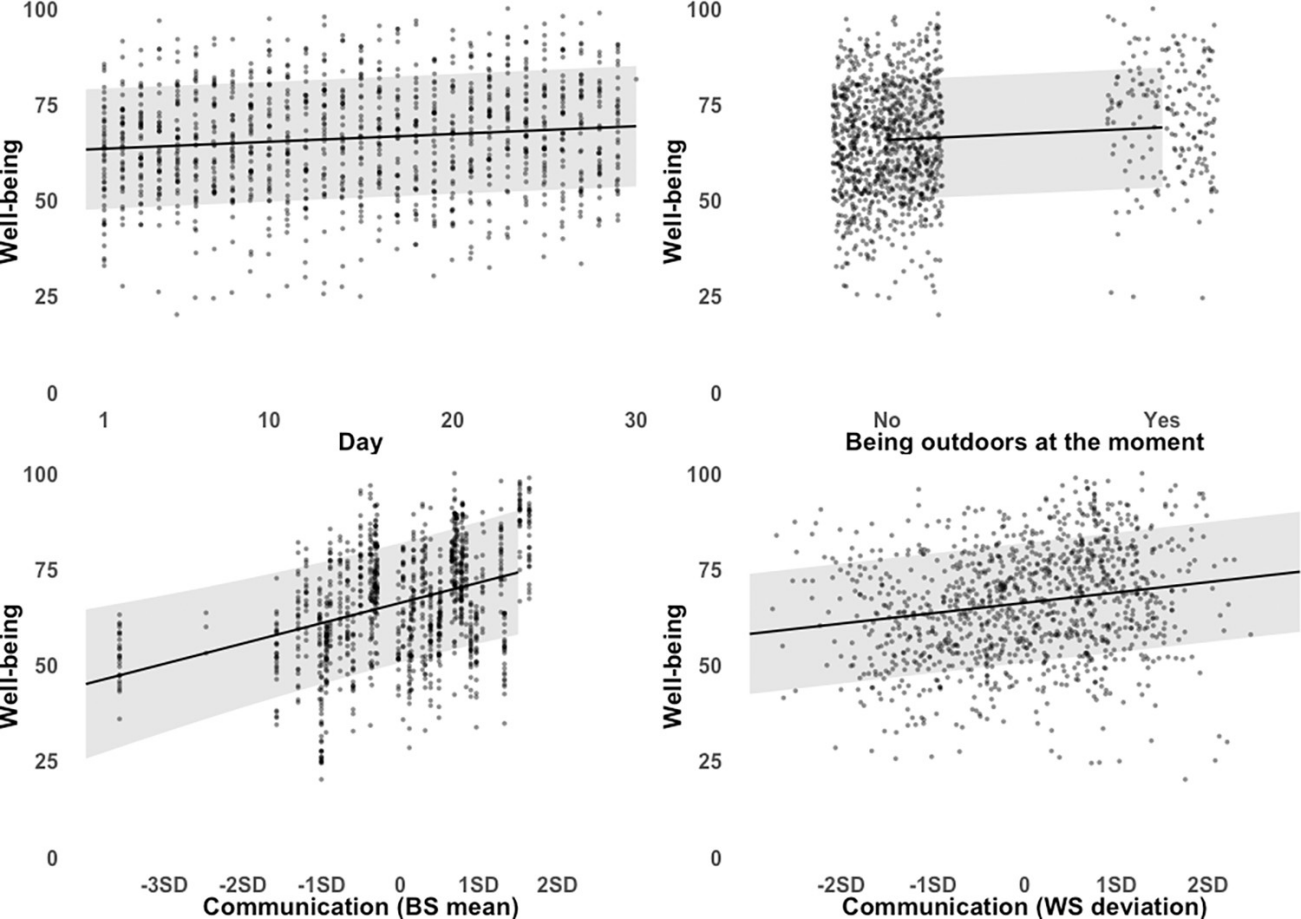
Predicted values of the well-being score for different values of day, being outdoors at the moment, and communication (between- and within-subject deviations). 95% confidence intervals are shown for predicted values (marginal effects) conditioned on fixed and random effects. Grey dots display the raw data points.

The results of the linear mixed-effects model indicate that the predictors explain 24% of the variance in the well-being score*; R*^*2*^_marginal_ = 0.24, *R*^*2*^_conditional_ = 0.72 (see Table 5). During the study, well-being improved over time, *b* = 0.20, *SE* = 0.07, *p* = .002. At the between-subjects level, participants who scored higher in perceived sufficiency of social contacts reported higher well-being on average, *b* = 0.36, *SE* = 0.10, *p* = .001. On a daily basis, more sufficient social contacts were associated with higher well-being, *b* = 0.12, *SE* = 0.01, *p* < .001. The participants who were outdoors at the time of survey response reported higher well-being than those who were not outdoors, *M*_*yes*_ = 71.76, *SD*_*yes*_ = 14.46, vs. *M*_*no*_ = 64.93, *SD*_*no*_ = 14.29, *b* = 3.29, *SE* = 0.68, *p* < .001. Being outdoors the day before the survey had no effect on well-being, *M*_*yes*_ = 66.23, *SD*_*yes*_ = 14.70, vs. *M*_*no*_ = 64.56, *SD*_*no*_ = 12.52, *b* = 1.26, *SE* = 0.89, *p* = .16. Neither the stringency index nor the number of new COVID-19 cases were associated with well-being, *b* = -0.38, *SE* = 0.34, *p* = .26 and *b* = -0.04, *SE* = 0.04, *p* = .39. The marginal effects of the predictors on the well-being score are shown in Fig 8.

**Table 5:** Regression coefficients for well-being score.

	Well-being		
*Predictors*	*Estimates*	*CI*	*p*
(Intercept)	90.93	40.38 – 141.49	<0.001
Being outdoors at the moment	3.29	1.96 – 4.61	<0.001
Being outdoors yesterday	1.26	-0.49 – 3.02	0.16
Day	0.20	0.07 – 0.33	0.002
Stringency index	-0.38	-1.05 – 0.29	0.26
New COVID-19 cases (in thousands)	-0.04	-0.12 – 0.05	0.39
Communication (BS mean)	0.36	0.15 – 0.57	0.001
Communication (WS deviation)	0.12	0.10 – 0.14	<0.001
Information (BS mean)	-0.08	-0.22 – 0.06	0.28
Information (WS deviation)	0.01	-0.03 – 0.05	0.53
Material resources (BS mean)	0.15	-0.08 – 0.38	0.20
Material resources (WS deviation)	0.03	-0.01 – 0.07	0.15
Random Effects			
σ^2^	61.20		
τ_00_ _participant_	118.81		
τ_11_ _participant x day_	0.14		
ρ_01_ _participant_	-0.47		
ICC	0.63		
N _participant_	46		
Observations	1112		
Marginal R^2^ / Conditional R^2^	0.24 / 0.72		

Additionally, we have tested an extended version of the model where the interactions between the stringency index, the number of new COVID-19 cases and the amount of communication (WS deviation) were included. The interactions were not statistically significant (*p*s > .05). We have also tested a regression model with fixed effects of each participant and each measurement week. The main findings remained the same. More sufficient social contacts were associated with higher well-being, *b* = 0.12, *SE* = 0.01, *p* < .001. The participants who were outdoors at the time of survey had higher well-being than the participants who were not outdoors, *b* = 3.12, *SE* = 0.72, *p* < .001, and well-being improved with each measurement week, *b* = 1.59, *SE* = 0.24, *p* < .001.

To understand which aspects of well-being were associated with the sufficiency of resources, we repeated the analysis for each item of the well-being score: life satisfaction, stress level, fear of getting infected, boredom, worries about significant others, and financial concerns (see Appendix B in S1 File for regression tables).

The perceived sufficiency of social contacts had a positive association with life satisfaction and a negative association with stress and boredom. At the between-subjects level, participants higher in the perceived sufficiency of social contacts were more satisfied with their lives, *b* = 0.51, *SE* = 0.15, *p* = .001, and were less stressed, *b* = -0.39, *SE* = 0.16, *p* = .019. On a daily basis, more sufficient social contacts were associated with a higher level of life satisfaction, *b* = 0.24, *SE* = 0.02, *p* < .001, and with a lower level of stress, *b* = -0.22, *SE* = 0.03, *p* < .001. The boredom was negatively related to the perceived sufficiency of social contacts both daily and between subjects. More sufficient social contacts on a particular day reduced boredom, *b* = -0.28, *SE* = 0.03, *p* < .001, and participants with a higher perceived sufficiency of social contacts were less bored on average, *b* = -0.69, *SE* = 0.11, *p* < .001.

The positive change in the perceived sufficiency of available daily material resources was associated with fewer financial concerns, *b* = -0.07, *SE* = 0.03, *p* = .007. At the between-subjects level, a higher level of perceived sufficiency of COVID-19 related information was associated with higher fear of getting infected by COVID-19, *b* = 0.31, *SE* = 0.15, *p* = .039. On the other hand, the fear declined each day, *b* = -0.54, *SE* = 0.15, *p* < .001. Worries about significant others getting infected also decreased during the study, *b* = -0.71, *SE* = 0.14, *p* < .001. Being outdoors at the moment of the survey was positively associated with life satisfaction, *b* = 4.33, *SE* = 1.24, *p* < .001, and lower level of boredom and stress, *b* = -5.20, *SE* = 1.58, *p* = .001, and *b* = -6.42, *SE* = 1.69, *p* < .001. Being outdoors one day before the measurement was positively associated with life satisfaction, *b* = 3.68, *SE* = 1.64, *p* = .025.

### Discussion

Study 2 aimed to explore how the changes in perception of the sufficiency of material, social, and informational resources are related to well-being during the COVID-19 pandemic. The data collected over four weeks allowed us to differentiate the relationships on two levels: 1) between-subjects differences (how is the average level of the sufficiency of resources related to the average level of well-being) and 2) within-subjects fluctuations (how is the change in the sufficiency of resources related to the changes in well-being).

We found that well-being improved with more sufficient social contacts. In particular, a higher subjective sufficiency of social contacts was associated with reduced stress and boredom and increased life satisfaction. These results are in line with previous research. Alfawaz and colleagues [52] found that people with a more intense family bond suffered less from anxiety and depression during the lockdown. Family bonding included spending more time with family members, e.g., cooking or exercising together at home. Having children at home during quarantine decreases the risk of depression [53].

Access to material resources such as household goods is generally associated with financial well-being. In our study, people worried more about potential financial losses when material resources were insufficient. Perhaps the sufficiency of household goods might serve as a proxy that indicates potentially more detrimental economic problems, e.g., loss of employment or salary reduction.

Being outdoors significantly improved the reported well-being in terms of higher life satisfaction and lower stress and boredom. This effect is in line with many other studies before COVID-19 that demonstrated the positive effect of being in natural environments on well-being [54]. Studies done during COVID-19 found that time spent outdoors was associated with increased positive emotions [55] and happiness [56]. Interestingly, being outdoors the day before the survey was positively related to life satisfaction but did not affect stress or boredom. These results suggest that the relationships between being outdoors and stress or boredom are correlative, but not causal.

As in any experience-sampling study, the well-being of participants could change during participation in the study. We observed two consistent trends over the measurement days: the fear to get infected and the worries about significant others getting infected were both decreasing over time. This coincides with the decrease of the stringency index in Germany during the study period, which means that the restrictions were loosening. This positive trend, however, partially contradicts the number of registered COVID-19 cases, which was increasing until the middle of April 2021, and only after that started to go down. However, we found no consistent effects of the stringency index or the number of new COVID-19 cases on fear or worries. Thus, the decline in fear and worries could be an effect of repeated measurements, such as some participants when asked repeatedly reported less fear and worries over time.

## General discussion

COVID-19 has changed people’s lives. Aside from the primary effects of the pandemic on population health, many of these changes were secondary—consequences of alterations in social regulations designed to prevent the spread of the virus. In this study, we wanted to understand how the restrictive measures affected people’s well-being. We approached this question from two different directions: One was a cross-sectional study that provided a snapshot of people’s lives during the pandemic. The second was an experience-sampling study that captured the dynamics of everyday life.

The results of both studies are consistent with a general “resource and demand” framework that we established in the introduction. This framework, rooted in common sense, is supported in psychology, in the theory of resource conservation [19]. Within this framework, all new social policies (e.g., wearing masks in public) are understood as demands. The response that these demands elicit in people depends on their ability to cope. Access to material resources, as well as non-material resources such as social contacts and information, is likely to increase adaptive capacity and well-being, and lead to a generally positive evaluation of the policy. On the other hand, lack or perceived loss of resources reduces adaptive capacity and well-being and is associated with a more negative policy evaluation.

### Policy implications

Support for restrictive policies is directly related to compliance—individuals who support restrictions (e.g., social distancing and mask-wearing) are more likely to comply. Previous research indicated that individual differences such as liberal political ideology, high levels of risk aversion, self-control, and need for cognition are associated with higher compliance [57]. Although these findings are potentially informative, we believe that they are only a part of the picture, and compliance should also be considered in the context of an individual’s ability to adapt to new policies. Some of these, such as wearing masks, are easier than others, such as quarantine. This individualized approach will also focus more on helping people build coping resources rather than individual differences, such as political opinion. As we found in Study 2, potential stress factors that can make people vulnerable to restrictive measures include financial loss, non-employment, and children in the household, so individuals who lost their jobs and income or have children at home may need additional support from the government to cope with the challenges of the pandemic.

One of the effective coping strategies in stressful times has been seeking social support coping [58]. In line with this, Study 2 demonstrated that well-being during COVID-19 was positively associated with the subjective sufficiency of social contacts. However, reducing social contacts was one of the main restrictive measures to stop the spread of the pandemic, e.g., canceling public gatherings and restricting movement. How can social contacts be supported without jeopardizing health? One way is to promote a “soft” version of social distancing, such as wearing masks with other people or meeting outdoors (which also has a positive effect on well-being, according to Study 2), without forcing strict self-isolation and avoiding any social contact at all. Previous studies have shown that the way the restrictions or regulations are conveyed to the public might play a role, as a more autonomous type of message (“You are in charge”) can produce less defiance than a controlling type of message based on shame and blame (“Do you want to kill another person?”) [59]. Martela et al. [60] proposed communication guidelines, which may help to promote compliance. For example, they recommended to emphasize a “shared identity and common fate” and “build trust through transparent and open communication”.

### Study limitations

There are two limitations of Study 2 related to the sample and measures. Study 2 was conducted with a sample of college students that may not be representative of the general population. Compared with Study 1, the sample of students was more homogenous in terms of their socio-demographic and economic characteristics, such as age, income, or living conditions. Although we focused on within-subject variation across multiple measurements, the effects observed from Study 2 might be different in the general population.

The other limitation concerns the operationalization of the dependent variable. In Study 2, we asked participants about the sufficiency of their resources. It could be that their responses reflected not only the objective amount of resources but other subjective evaluations of their current situations, such as general optimism. A more rigorous test of the idea that social communication relates to well-being will be to ask participants to report about social contacts they have engaged in. Another potential problem is that some other variable can account for the positive correlation between the sufficiency of social contacts and well-being. For example, a physical condition (being healthy vs. sick) might be related both to the sufficiency of social contacts and well-being. We did not ask participants to report their physical condition daily. However, we controlled for the variables related to being outdoors at the moment and the day before the survey, which can serve as a proxy for their physical health (based on the expectation that physically ill people would mainly remain at home without going outside).

### Future research

Future studies could explore the relationship between the content of consumed COVID-19-related information and well-being. We assume that there are two types of informational content that have different effects on people’s well-being. One is positive news and updates about new developments, technologies, and measures that may relieve stress and improve well-being. The other is distressing news that reports shocking events, dramatic half-truths, and horrid future scenarios and induces fear. This idea originates from another study by our research group, which compared COVID-19-related attitudes in Germany and Switzerland and found systematic differences in news sources and COVID-19-related fear between the two countries [61].

The theory of stress postulates the perceived inability to cope with high demands as the main cause of stress [17]. Future studies may measure perceived ability to cope with restrictive measures as a potential mediator between the measures’ intensity and stress level.

An experience sampling study offers insight into everyday life that can reveal its dynamics. Future studies could combine it with interventions that further test causal relationships. In particular, the intervention related to social contacts and access to information could be interesting to study. The potential question would be how the quality of social contacts or information consumed affects well-being during the lockdown time. Another perspective is to explore the moderating effect of personality, e.g., introversion vs. extroversion.

Longitudinal studies with the experience sampling method are sensitive to social context. Future studies could incorporate the survey of perceptions of changing regulations to find out how people adjust their attitudes and behavior in response to changes in policy.

## Supporting information

S1 File (DOCX)
