# Supplementary material for: Psychological well-being during the COVID-19 pandemic: Combining a web survey with experience sampling methodology
Source: PLoS One. 2023 Mar 24;18(3):e0282649. doi: 10.1371/journal.pone.0282649 (PMC10038280; doi:10.1371/journal.pone.0282649)
Supplement: S1 File — (DOCX) [file pone.0282649.s001.docx]

Appendix A

**Table A1. Definitions and Summary Statistics of the Variables in Study 1.**

| Variable | Survey question | Number of responses | | | |
| --- | --- | --- | --- | --- | --- |
| Financial loss^a^ | Did you experience financial losses due to the coronavirus pandemic and its consequences? | *N_No loss_* = 408 (60%), *N_Up to one_* = 106 (15%), *N_More than one_* = 163 (24%), *N_No response_* = 8 (1%) | | | |
| Home pet | Do you have any pets (not farm animals)? | *N_No_* = 349 (51%), *N_Yes_* = 313 (46%), *N_No response_* = 23 (3%) | | | |
| Self-isolation^b^ | Have you done self-isolation for at least 7 days? | *N_No_* = 581 (85%), *N_Yes_* = 104 (15%) | | | |
|  | | Mean | Median | SD | Range |
| Number of children^c^ | How many children live in your household? | 1.66 | 2 | 0.78 | 1 - 6 |
| Income level^d^ | Please provide information about your annual income (before taxes). Please provide your best estimates. | 3.63 | 4 | 1.75 | 1 - 8 |
| Frustration | How frustrated do you feel in the current moment? | 68.47 | 80.00 | 35.20 | 0 - 100 |
| Life satisfaction | All things considered, how satisfied are you with your life in general at present? | 5.18 | 5 | 2.65 | 1 - 10 |
| Information access^e^ | How frequently do you perceive information on the Corona virus through the media? | 4.42 | 5 | 1.08 | 1 - 5 |
| Rooms per person^f^ | How many rooms are available to the household? With how many people do you live in your household? | 1.91 | 1.67 | 0.88 | 0.5 – 8 |
| COVID-19 statistics^g^ | | | | | |
| Stringency index | | 58.91 | 60.65 | 7.76 | 37.04 – 85.19 |
| New COVID-19 cases (in thousands) | | 11.60 | 11.52 | 7.24 | 0.22 – 66.88 |

1. Response options were “No loss”, “Up to one my month income” and “More than one my month income”.
2. The participants were provided with the following definition of self-isolation – “According to the National Health Service of UK, the self-isolation means “do not leave your home if you have symptoms of coronavirus (COVID-19) or live with someone who does”. If you are self-isolating, you must: 1) not leave your home for any reason, other than to exercise once a day – but stay at least 2 meters (3 steps) away from other people 2) not go out to buy food or collect medicine – order them by phone or online, or ask someone else to drop them off at your home 3) not have visitors, such as friends and family, in your home”.
3. The summary statistics was computed for participants who indicated that they had at least one child. 407 participants indicated that they did not have children. Among 272 participants who had children, there were one child (*n* = 131), two children (*n* = 111), three (*n* = 26), four (*n* = 2), and six (*n* = 2).
4. Response options were coded as 1 – < €15,000 (*n* = 87); 2 – €15,001- €25,000 (*n* = 107); 3 – €25,001- €35,000 (*n* = 114); 4 – €35,001- €50,000 (*n* = 163); 5 – €50,001- €75,000 (*n* = 107); 6 – €75,001- €100,000 (*n* = 44); 7 – €100,001- €150,000 (*n* = 28); 8 – > €150,000 (*n* = 16).
5. Response options were coded as 1 – Less than once a week (*n* = 37); 2 – Once a week (*n* = 9); 3 – Several times a week (*n* = 62); 4 – Once a day (*n* = 95); 5 – Several times a day (*n* = 478).
6. The number of rooms per person was calculated by dividing the number of rooms by the number of people in the household. The number of rooms and people were asked with the questions “How many rooms are available to the household?” and “With how many people do you live in your household?”.
7. The stringency index and the number of new COVID-19 cases per day were obtained from the database at <https://ourworldindata.org/grapher/covid-stringency-index>.

**Table A2. Summary Statistics of the Variables for Subgroups in Study 1.**

The subgroups are based on the answer to the question “Did you experience financial losses due to the coronavirus pandemic and its consequences?” and response options “No loss”, “Up to one my month income” and “More than one my month income”.

| Variable | The subgroup | | | Statistical test of group differences |
| --- | --- | --- | --- | --- |
|  | No loss  (*n* = 408) | Less than one month income  (*n* = 106) | More than one month income (*n* = 163) |  |
| Gender | 270 women (67%) | 65 women (58%) | 94 women (63%) | *X^2^* (2, *N* = 671) = 4.34, *p* = .11 |
| Age | 46 (11.15) | 44 (10.45) | 46 (10.51) | *F*(2, 668) = 1.88, *p* = .15 |
| Education level | 2.30 (0.62) | 2.16 (0.68) | 2.26 (0.67) | *F*(2, 668) = 1.80, *p* = .17 |
| Income level | 3.74 (1.72) | 3.64 (1.76) | 3.41 (1.79) | *F*(2, 656) = 2.04, *p* = .13 |
| Non-employment | *N* = 42 (10%) | *N* = 13 (13%) | *N* = 45 (28%) | *X^2^* (2, *N* = 668) = 28.36, *p* < .001 |
| Rooms per person | 1.97 (0.91) | 1.86 (0.87) | 1.78 (0.80) | *F*(2, 667) = 2.96, *p* = .052 |
| Children | 0.61 (0.91) | 0.72 (0.91) | 0.78 (1.07) | *F*(2, 668) = 2.12, *p* = .12 |
| Home pet | 183 pet owners (46%) | 42 pet owners (42%) | 84 pet owners (53%) | *X^2^* (2, *N* = 655) = 3.17, *p* = .20 |
| Information access | 4.41 (1.06) | 4.53 (1.01) | 4.41 (1.14) | *F*(2, 670) = 0.57, *p* = .57 |
| Stringency index | 58.47 (8.14) | 58.64 (8.02) | 59.99 (6.56) | *F*(2, 674) = 2.28, *p* = .10 |
| New COVID-19 cases (in thousands) | 11.23 (7.40) | 11.24 (6.77) | 12.64 (7.01) | *F*(2, 674) = 2.38, *p* = .09 |

**Table A3. Regression Coefficients for Stress and Support.**

|  | Stress by restrictive measures | | | Support for restrictive measures | | |
| --- | --- | --- | --- | --- | --- | --- |
| *Predictors* | *Estimates* | *CI* | *p* | *Estimates* | *CI* | *p* |
| (Intercept) | 36.90 | 28.98 – 44.81 | **<0.001** | 52.08 | 43.45 – 60.70 | **<0.001** |
| Financial loss  (less than one month income) | 10.91 | -5.36 – 27.19 | 0.19 | -8.81 | -26.54 – 8.93 | 0.33 |
| Financial loss  (more than one month income) | 27.36 | 16.36 – 38.37 | **<0.001** | -33.67 | -45.66 – -21.68 | **<0.001** |
| Employment | 10.32 | 1.95 – 18.68 | **0.016** | -13.43 | -22.54 – -4.32 | **0.004** |
| Employment x  Financial loss  (less than one month income) | 1.00 | -16.36 – 18.35 | 0.91 | -3.30 | -22.21 – 15.61 | 0.73 |
| Employment x  Financial loss  (more than one month income) | -15.26 | -27.55 – -2.97 | **0.015** | 16.76 | 3.37 – 30.15 | **0.014** |
| Observations | 668 | | | 668 | | |
| R^2^ / R^2^ adjusted | 0.07 / 0.07 | | | 0.10 / 0.09 | | |

**Table A4. Definitions and Summary Statistics of the Variables in Study 2.**

| Variable | Survey question | Summary statistics | | | |
| --- | --- | --- | --- | --- | --- |
| Baseline survey | | | | | |
|  |  | Number of responses | | | |
| Financial loss^a^ | Did you experience financial losses due to the coronavirus pandemic and its consequences? | *N_No loss_* = 39 (85%), *N_Up to one_* = 4 (9%), *N_More than one_* = 3 (6%) | | | |
| Home pet | Do you have any pets (not farm animals)? | *N_No_* = 40 (87%), *N_Yes_* = 6 (13%) | | | |
| Self-isolation^b^ | Have you done self-isolation for at least 7 days? | *N_No_* = 35 (76%), *N_Yes_* = 11(24%) | | | |
|  | | Mean | Median | SD | Range |
| Income level^c^ | Please provide information about your annual income (before taxes). Please provide your best estimates. | 1.16 | 1 | 0.90 | 1 – 7 |
| Frustration | How frustrated do you feel in the current moment? | 57.78 | 63.50 | 25.27 | 0 – 98 |
| Information access^d^ | How frequently do you perceive information on the Corona virus through the media? | 4.04 | 4 | 0.99 | 2 – 5 |
| Rooms per person^e^ |  | 1.22 | 1 | 0.59 | 0.1 – 3 |
| *Daily measures^f^* | | | | | |
| Being outdoors at the moment^g^ | Where are you right now? | 0.16 | 0.15 | 0.11 | 0 – 0.57 |
| Being outdoors the day before | Have you been outside yesterday? | 0.89 | 0.96 | 0.18 | 0 - 1 |
| Communication | How far is your today’s social communication sufficient for your normal level? | 60.79 | 63.18 | 14.46 | 9.11 – 84.31 |
| Information | To what extent is the level of information about the current situation related to COVID-19 (e.g., guidance from the authorities, news) you receive sufficient? | 58.28 | 65.08 | 22.11 | 6.80 – 96.24 |
| Material resources | To what extent is your today’s basic supply (e.g. food, water, household goods) sufficient? | 85.40 | 88.00 | 13.36 | 47.80 – 96.60 |
| Life satisfaction | How satisfied are you with your life at the current moment? | 62.42 | 64.12 | 15.41 | 16.21 – 91.56 |
| Stress level | How stressed do you feel at the current moment? | 40.93 | 41.91 | 15.78 | 5.50 – 85.41 |
| Fear to get infected | How much do you fear to be infected at the current moment? | 33.63 | 27.12 | 21.28 | 0.80 – 75.89 |
| Boredom | How much are you bored at the current moment? | 31.85 | 29.18 | 14.96 | 1.35 – 69.29 |
| Worries about significant others | How worried are you at this moment that your loved ones might be infected? | 39.78 | 35.37 | 23.99 | 1.00 – 84.58 |
| Financial concerns | How concerned are you about your potential economic loss at this moment? | 18.83 | 12.98 | 18.91 | 0.04 – 70.21 |
| COVID-19 statistics^h^ | | | | | |
| Stringency index | | 73.56 | 75.00 | 3.03 | 67.60 – 77.78 |
| New COVID-19 cases (in thousands) | | 11.73 | 10.80 | 8.28 | 0.21 – 29.52 |

1. Response options were “No loss”, “Up to one my month income” and “More than one my month income”.
2. The participants were provided with the following definition of self-isolation – “According to the National Health Service of UK, the self-isolation means “do not leave your home if you have symptoms of coronavirus (COVID-19) or live with someone who does”. If you are self-isolating, you must: 1) not leave your home for any reason, other than to exercise once a day – but stay at least 2 meters (3 steps) away from other people 2) not go out to buy food or collect medicine – order them by phone or online, or ask someone else to drop them off at your home 3) not have visitors, such as friends and family, in your home”.
3. Response options were coded as 1 – < €15,000 (*n* = 43); 2 – €15,001- €25,000 (*n* = 1); 3 – €25,001- €35,000 (*n* = 0); 4 – €35,001- €50,000 (*n* = 0); 5 – €50,001- €75,000 (*n* = 0); 6 – €75,001- €100,000 (*n* = 0); 7 – €100,001- €150,000 (*n* = 1); 8 – > €150,000 (*n* = 0).
4. Response options were coded as 1 – Less than once a week (*n* = 0); 2 – Once a week (*n* = 3); 3 – Several times a week (*n* = 12); 4 – Once a day (*n* = 11); 5 – Several times a day (*n* = 20).
5. The number of rooms per person was calculated by dividing the number of rooms by the number of people in the household. The number of rooms and people were asked with the questions “How many rooms are available to the household?” and “With how many people do you live in your household?”.
6. The daily survey items were measured with a 101-point visual analogue scale. To compute the summary statistics, the individual averages were first calculated across all days.
7. Response options were coded as 0 - Inside; 1 – Outside.
8. The stringency index and the number of new COVID-19 cases per day were obtained from the database at <https://ourworldindata.org/grapher/covid-stringency-index>.

Appendix B

**Table B1. Regression Coefficients for Life Satisfaction and Stress.**

|  | Life satisfaction | | | Stress level | | |
| --- | --- | --- | --- | --- | --- | --- |
| *Predictors* | *Estimates* | *CI* | *p* | *Estimates* | *CI* | *p* |
| (Intercept) | 108.35 | 36.63 – 180.06 | **0.003** | -11.85 | -125.97 – 102.27 | 0.84 |
| Being outdoors at the moment | 4.33 | 1.91 – 6.76 | **<0.001** | -6.42 | -9.75 – -3.10 | **<0.001** |
| Being outdoors yesterday | 3.68 | 0.46 – 6.89 | **0.025** | -2.24 | -6.63 – 2.16 | 0.32 |
| Day | -0.05 | -0.19 – 0.10 | 0.51 | 0.10 | -0.20 – 0.40 | 0.51 |
| Stringency index | -0.65 | -1.60 – 0.30 | 0.18 | 0.71 | -0.80 – 2.23 | 0.36 |
| New COVID-19 cases (in thousands) | -0.04 | -0.18 – 0.11 | 0.61 | 0.08 | -0.13 – 0.29 | 0.45 |
| Communication (BS mean) | 0.51 | 0.21 – 0.80 | **0.001** | -0.39 | -0.71 – -0.07 | **0.019** |
| Communication (WS deviation) | 0.24 | 0.20 – 0.28 | **<0.001** | -0.22 | -0.28 – -0.17 | **<0.001** |
| Information (BS mean) | 0.08 | -0.13 – 0.28 | 0.45 | -0.02 | -0.24 – 0.20 | 0.84 |
| Information (WS deviation) | 0.05 | -0.01 – 0.12 | 0.12 | -0.05 | -0.14 – 0.05 | 0.34 |
| Material resources (BS mean) | 0.14 | -0.19 – 0.47 | 0.40 | 0.06 | -0.30 – 0.42 | 0.74 |
| Material resources (WS deviation) | 0.01 | -0.06 – 0.08 | 0.83 | -0.06 | -0.15 – 0.04 | 0.25 |
| Random Effects | | | | | | |
| σ^2^ | 206.47 | | | 400.91 | | |
| τ_00_ _participant_ | 188.27 | | | 400.20 | | |
| τ_11_ _participant x day_ | 0.07 | | | 0.68 | | |
| ρ_01_ _participant_ | -0.18 | | | -0.72 | | |
| ICC | 0.48 | | | 0.39 | | |
| N _participant_ | 46 | | | 46 | | |
| Observations | 1112 | | | 1112 | | |
| Marginal R^2^ / Conditional R^2^ | 0.22 / 0.59 | | | 0.11 / 0.45 | | |

**Table B2. Regression Coefficients for Fear and Boredom.**

|  | Fear to get infected | | | Boredom | | |
| --- | --- | --- | --- | --- | --- | --- |
| *Predictors* | *Estimates* | *CI* | *p* | *Estimates* | *CI* | *p* |
| (Intercept) | 45.34 | -52.76 – 143.45 | 0.37 | 59.62 | -29.75 – 148.99 | 0.19 |
| Being outdoors at the moment | -0.72 | -3.09 – 1.65 | 0.55 | -5.20 | -8.29 – -2.10 | **0.001** |
| Being outdoors yesterday | 0.66 | -2.49 – 3.80 | 0.68 | -2.54 | -6.61 – 1.53 | 0.22 |
| Day | -0.54 | -0.83 – -0.25 | **<0.001** | -0.10 | -0.29 – 0.09 | 0.29 |
| Stringency index | -0.05 | -1.35 – 1.25 | 0.94 | -0.34 | -1.53 – 0.85 | 0.57 |
| New COVID-19 cases (in thousands) | -0.07 | -0.22 – 0.08 | 0.39 | 0.14 | -0.04 – 0.32 | 0.14 |
| Communication (BS mean) | -0.32 | -0.76 – 0.11 | 0.14 | -0.69 | -0.91 – -0.47 | **<0.001** |
| Communication (WS deviation) | 0.03 | -0.01 – 0.07 | 0.13 | -0.28 | -0.33 – -0.23 | **<0.001** |
| Information (BS mean) | 0.31 | 0.02 – 0.61 | **0.039** | 0.05 | -0.10 – 0.20 | 0.52 |
| Information (WS deviation) | -0.06 | -0.13 – 0.00 | 0.06 | 0.07 | -0.01 – 0.15 | 0.11 |
| Material resources (BS mean) | -0.20 | -0.69 – 0.29 | 0.41 | -0.11 | -0.35 – 0.14 | 0.39 |
| Material resources (WS deviation) | -0.05 | -0.12 – 0.02 | 0.19 | 0.03 | -0.06 – 0.12 | 0.53 |
| Random Effects | | | | | | |
| σ^2^ | 194.96 | | | 332.01 | | |
| τ_00_ _participant_ | 565.68 | | | 65.60 | | |
| τ_11_ _participant x day_ | 0.76 | | | 0.14 | | |
| ρ_01_ _participant_ | -0.53 | | | 0.22 | | |
| ICC | 0.71 | | | 0.27 | | |
| N _participant_ | 46 | | | 46 | | |
| Observations | 1112 | | | 1111 | | |
| Marginal R^2^ / Conditional R^2^ | 0.11 / 0.74 | | | 0.27 / 0.46 | | |

**Table B3. Regression Coefficients for Worries and Concern.**

|  | Worries about significant others | | | Financial concerns | | |
| --- | --- | --- | --- | --- | --- | --- |
| *Predictors* | *Estimates* | *CI* | *p* | *Estimates* | *CI* | *p* |
| (Intercept) | -7.73 | -101.11 – 85.66 | 0.87 | 81.13 | 5.98 – 156.28 | **0.034** |
| Being outdoors at the moment | -1.76 | -3.95 – 0.42 | 0.11 | -0.23 | -2.01 – 1.55 | 0.80 |
| Being outdoors yesterday | 0.82 | -2.07 – 3.72 | 0.58 | -0.16 | -2.52 – 2.19 | 0.89 |
| Day | -0.71 | -0.98 – -0.44 | **<0.001** | -0.10 | -0.32 – 0.11 | 0.36 |
| Stringency index | 0.77 | -0.47 – 2.01 | 0.22 | -0.81 | -1.81 – 0.18 | 0.11 |
| New COVID-19 cases (in thousands) | -0.03 | -0.17 – 0.10 | 0.63 | -0.01 | -0.12 – 0.10 | 0.89 |
| Communication (BS mean) | -0.24 | -0.75 – 0.27 | 0.35 | -0.11 | -0.49 – 0.28 | 0.59 |
| Communication (WS deviation) | 0.00 | -0.03 – 0.04 | 0.89 | -0.01 | -0.03 – 0.02 | 0.73 |
| Information (BS mean) | 0.14 | -0.21 – 0.48 | 0.43 | 0.20 | -0.06 – 0.47 | 0.13 |
| Information (WS deviation) | 0.02 | -0.05 – 0.08 | 0.63 | 0.00 | -0.05 – 0.05 | 0.99 |
| Material resources (BS mean) | -0.12 | -0.69 – 0.45 | 0.67 | -0.22 | -0.66 – 0.22 | 0.32 |
| Material resources (WS deviation) | -0.01 | -0.08 – 0.05 | 0.71 | -0.07 | -0.12 – -0.02 | **0.007** |
| Random Effects | | | | | | |
| σ^2^ | 166.19 | | | 108.77 | | |
| τ_00_ _participant_ | 651.49 | | | 353.91 | | |
| τ_11_ _participant x day_ | 0.67 | | | 0.42 | | |
| ρ_01_ _participant_ | -0.35 | | | -0.24 | | |
| ICC | 0.79 | | | 0.78 | | |
| N _participant_ | 46 | | | 46 | | |
| Observations | 1111 | | | 1111 | | |
| Marginal R^2^ / Conditional R^2^ | 0.08 / 0.81 | | | 0.05 / 0.79 | | |
